# Bundibugyo Virus Disease: Diagnostics and Medical Countermeasures for a Neglected Ebolavirus

**DOI:** 10.3390/v18070775

**Published:** 2026-07-15

**Authors:** Katharina Kopp

**Affiliations:** One Health Pathogenomics Consulting, 80687 Munich, Germany; koppk@onehealth-pathogenomics-consulting.org

**Keywords:** Bundibugyo virus disease, *Orthoebolavirus bundibugyoense*, filoviruses, diagnostics, antivirals, antibodies, vaccines, post-exposure prophylaxis, neglected tropical disease, viral hemorrhagic fever

## Abstract

Bundibugyo virus disease, caused by Bundibugyo virus (*Orthoebolavirus bundibugyoense*), is a severe human Ebola disease with substantial mortality, unresolved reservoir ecology, limited diagnostic implementation, and no licensed vaccines or therapeutics specifically approved for this *Orthoebolavirus* species. The May 2026 public health emergency in the Democratic Republic of the Congo and Uganda renewed the need for a focused synthesis of Bundibugyo virus-specific diagnostics and medical countermeasures. This review synthesizes the peer-reviewed literature, preprints, and official public-health documents on diagnostics, antivirals, therapeutics, vaccines, and post-exposure prophylaxis. Comparative evidence from the Ebola virus, Sudan virus, Marburg virus, and pan-filovirus platforms is included only where it clarifies Bundibugyo virus-specific evidence, exposes unsupported extrapolation, or defines preparedness gaps. The 2007–2008 outbreak showed that assays optimized for known filoviruses can miss divergent ebolaviruses; the 2026 outbreak underscored the importance of diagnostic breadth, sequencing-based confirmation, decentralized laboratory capacity, and regional coordination. Clinical and immunological data indicate that Bundibugyo virus cannot be reduced to an Ebola virus-like model. Countermeasure evidence remains largely preclinical: recombinant vesicular stomatitis virus vaccines expressing Bundibugyo virus glycoprotein provide the strongest direct animal protection data, whereas antiviral and antibody-based evidence varies widely and requires careful separation of direct Bundibugyo virus data from platform-based extrapolation.

## 1. Introduction

Bundibugyo virus (BDBV) is an *Orthoebolavirus* assigned to the species *Orthoebolavirus bundibugyoense* in the family *Filoviridae* [1]. Under contemporary filovirus disease terminology, disease caused by BDBV is best referred to as Bundibugyo virus disease (BVD) or Ebola disease caused by BDBV, whereas Ebola virus disease in the strict sense refers to disease caused by Ebola virus (EBOV), assigned to *Orthoebolavirus zairense* [1,2,3]. This distinction is not merely semantic. Species-level differences among ebolaviruses affect diagnostic target choice, antigenic coverage, animal-model behavior, vaccine design, therapeutic breadth, and interpretation of medical countermeasure evidence.

**Figure 1:**
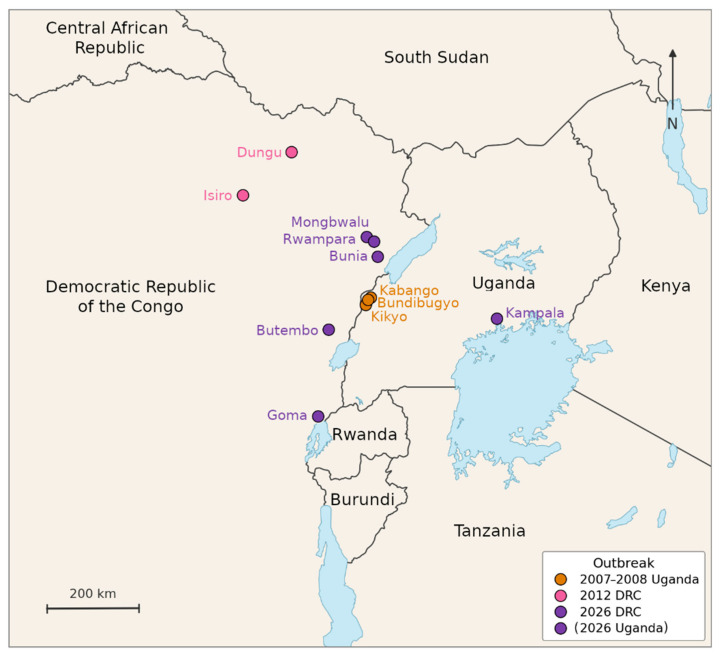
Geographic distribution of recognized Bundibugyo virus disease outbreak locations. Dots indicate approximate outbreak-recognition, reported-affected, or index-associated locations, not transmission routes or outbreak-area boundaries. The 2007–2008 Uganda outbreak is represented by Kikyo, Kabango, and Bundibugyo [4,5,6,7]. The 2012 Democratic Republic of the Congo (DRC) outbreak is represented by Isiro and Dungu [9,10,11]. The 2026 outbreak is represented by Mongbwalu, Rwampara, Bunia, Butembo, and Goma in the DRC, and Kampala, Uganda, where imported cases from the DRC were reported (as of 18 May 2026) [12,17,18,19,20,21,22].

The first recognized BVD outbreak occurred in Bundibugyo District, western Uganda, during August 2007–February 2008, with Kikyo, Kabango, and Bundibugyo shown in Figure 1 [4,5,6,7]. The putative index patient in the epidemiological case-series investigation was a 26-year-old woman from Kabango village, Kasitu subcounty. She was hospitalized on 1 August 2007, delivered a preterm infant the following day, developed diarrhea and difficulty breathing without hemorrhagic manifestations, and died on 4 August [5]. The initial cluster associated with this patient included nine case patients and six deaths, corresponding to a 67% case-fatality rate [5].

In the full outbreak investigation, 192 persons met the suspected-case definition; 42 were laboratory-confirmed, 74 remained probable, and 76 were laboratory-negative and classified as non-cases [5]. Overall, 39 of 116 confirmed or probable cases died, corresponding to a 34% case-fatality rate, while the rate among confirmed cases was 33% [5]. The Centers for Disease Control and Prevention (CDC) outbreak-history summary lists 131 reported cases and 42 deaths for the 2007 Uganda outbreak, corresponding to a 32% case-fatality rate [7]. These differences reflect different case definitions and reporting denominators rather than a single uniform dataset [5,6,7,8]. Among 26 hospitalized laboratory-confirmed patients with detailed clinical data, the most frequent symptoms included non-bloody diarrhea, severe headache, asthenia, nausea or vomiting, myalgia, abdominal pain, dysphagia, and appetite loss. Hemorrhagic manifestations were recorded in seven patients, six of whom died [6].

The second recognized BVD outbreak occurred in 2012 in northeastern Democratic Republic of the Congo (DRC), centered on the Isiro Health Zone in the former Orientale Province, with Isiro and Dungu shown in Figure 1 [9,10,11]. The definitive index case was not identified. The earliest polymerase chain reaction (PCR)-confirmed BDBV-containing sample described in the retrospective molecular analysis came from a clinic nurse in Isiro whose disease onset was estimated as 28 June 2012. This patient had several possible exposures, including direct contact with sick persons, funeral attendance, and exposure to bats [11].

The 2012 outbreak resulted in 38 laboratory-confirmed cases and 13 deaths, corresponding to a 34% case-fatality rate among laboratory-confirmed cases [7,11]. A broader clinical dataset that included probable and suspected cases reported 62 cases and 34 deaths, corresponding to a 46.8% case-fatality rate [10,11]. Retrospective sequencing expanded the available genomic dataset and challenged a simple single-introduction interpretation by identifying evidence compatible with earlier BDBV circulation and more than one spillover or introduction scenario [11]. The World Health Organization (WHO) summarizes the case-fatality rates of the previous two BVD outbreaks as approximately 30–50% [12]. BDBV should therefore not be described as a mild or marginal ebolavirus. Rather, it is a recurrent human ebolavirus for which the diagnostic and medical countermeasure evidence base is much smaller than that for EBOV.

Bundibugyo virus has usually been addressed within broader reviews of Ebola disease, filovirus genomics, ebolavirus virulence, vaccines, or therapeutics [3,13,14,15,16]. These reviews provide essential context, but they do not synthesize BDBV-specific diagnostic recognition, small-molecule antiviral evidence, antibody-, protein-, peptide-, and nucleic-acid-based therapeutics, vaccines, and post-exposure prophylaxis as a single preparedness problem. To our knowledge, no dedicated review has evaluated BDBV diagnostics and medical countermeasures across these categories. The current outbreak and the absence of licensed BDBV-specific vaccines or therapeutics make this gap operationally relevant rather than merely taxonomic.

This review prioritizes studies containing direct BDBV diagnostic, clinical, immunological, animal-model, antiviral, antibody, vaccine, or post-exposure data. Evidence is interpreted by category: direct human BVD data; authentic BDBV challenge data from nonhuman primates (NHPs), ferrets, or other animal models; authentic BDBV in vitro data; BDBV surrogate-virus animal data; BDBV glycoprotein (GP)-pseudotyped or minigenome data; BDBV antigen-inclusion data; BDBV epitope-focused design evidence; extrapolation from other filoviruses; and official outbreak or preparedness documentation. Virus and taxon names follow current International Committee on Taxonomy of Viruses (ICTV) taxonomy, while disease terminology follows contemporary filovirus disease nomenclature recommendations [1,2]. Preprints and official public-health documents were used where appropriate for diagnostic, outbreak, sequencing, coordination, or operational preparedness context, but not as efficacy evidence unless the underlying study design supported such interpretation.

During the May 2026 BVD public health emergency in the DRC and Uganda, the Ministry of Public Health, Hygiene and Social Welfare of the DRC declared the country’s 17th Ebola disease outbreak, affecting the Rwampara, Mongbwalu, and Bunia health zones in Ituri Province (Figure 1) [17]. The WHO was alerted on 5 May 2026 to a high-mortality illness in Mongbwalu Health Zone, including deaths among health workers [12]. The earliest suspected case described in the WHO disease outbreak notice was a health worker with symptom onset on 24 April 2026 who died at a medical center in Bunia [12]. On 14 May 2026, the Institut National de Recherche Biomédicale (INRB) analyzed 13 blood samples from the Rwampara Health Zone; BVD was confirmed in eight samples on 15 May 2026 [12].

The WHO subsequently determined that Ebola disease caused by BDBV in the DRC and Uganda constituted a public health emergency of international concern [18]. Reported counts evolved rapidly. The WHO statement published on 17 May 2026 reported eight laboratory-confirmed cases, 246 suspected cases, and 80 suspected deaths in the Ituri Province as of 16 May 2026 [12,18]. The CDC 19 May 2026 situation report, citing the DRC and Uganda Ministries of Health, subsequently reported 536 suspected cases, 105 probable cases, 34 confirmed cases, and 134 deaths across the DRC and Uganda [19]. On 20 May 2026, Reuters, citing WHO officials, reported 600 suspected cases, 139 suspected deaths, 51 laboratory-confirmed cases in the DRC, and two confirmed cases in Uganda; the same report stated that investigations were ongoing and that transmission may have begun approximately two months before formal confirmation [20].

The CDC also reported that the two BVD cases identified in Uganda were not locally acquired, but occurred in persons who had traveled to Kampala from the DRC; one patient died, and no onward transmission had been reported in Uganda [19]. The DRC outbreak had been reported in nine health zones in the Ituri Province as of 18 May 2026 [20]. Africa Centres for Disease Control and Prevention (Africa CDC) responded as the African Union’s regional public-health authority, convening urgent regional coordination with the DRC, Uganda, South Sudan, WHO, and other partners [21]. Africa CDC highlighted the risk of further spread related to urban transmission, mining-associated mobility, insecurity, infection-prevention gaps, and cross-border movement [21]. BEACON event reports subsequently recorded additional outbreak-tracking information, including Goma as a reported DRC location and an unspecified preliminary positive test in South Sudan [22]. South Sudan should be treated as preliminary event-tracking information unless confirmed in WHO, CDC, Africa CDC, or national Ministry of Health reporting. Subsequent WHO-convened advisory meetings identified candidate therapeutics and vaccines for clinical-trial evaluation during the 2026 BVD outbreak, while emphasizing that no vaccine or therapeutic was licensed specifically for BVD and that the identified products should be used exclusively within clinical trials [23].

The 2007–2008 BDBV outbreak provides the historical precedent for a diagnostic vulnerability that remains central to BVD preparedness: target mismatch can occur when assays are optimized for known filoviruses but the causative ebolavirus species is unknown. A divergent ebolavirus was identified only after initial assays optimized for then-known filoviruses were negative and broader testing with sequencing was pursued [4]. This episode illustrates why BVD preparedness requires diagnostic workflows that can escalate beyond species-focused assays when the causative ebolavirus is unknown.

The May 2026 outbreak showed that this vulnerability persists. Initial real-time reverse transcription quantitative PCR (RT-qPCR) testing performed with an EBOV-focused cartridge kit was negative, whereas subsequent broader testing at the Institut National de Recherche Biomédicale (INRB) confirmed *Orthoebolavirus* infection and genomic sequencing identified BDBV [12,19]. This sequence illustrates the limitation of species-focused workflows when the causative ebolavirus is unknown. It does not indicate a general failure of cartridge-based diagnostics; rather, it shows that EBOV-directed negative results should prompt broader testing and sequencing when clinical and epidemiological suspicion for filovirus disease remains high [12,19,24,25].

Preparedness for BVD also extends beyond acute outbreak detection and survival. Long-term follow-up of BVD survivors from the 2007–2008 outbreak in Uganda identified ocular deficits, hearing loss, difficulty swallowing, sleep disturbance, arthralgia, fatigue, constitutional symptoms, chronic health problems, and memory or confusion-related limitations more than two years after infection [26]. A subsequent BVD-specific cross-sectional study of 40 laboratory-confirmed survivors and 23 controls 16 years after infection reported persistent multisystem sequelae, including neurological and musculoskeletal complaints, headaches, visual disturbances, and stigma [27]. These findings support survivor follow-up and longitudinal clinical care in the broader BVD preparedness framework, although they do not directly inform acute diagnostic performance or countermeasure efficacy. Direct BDBV-specific evidence for persistent infectious virus after recovery, relapse, semen shedding, sexual transmission, or treatment of persistent infection was not identified. Addressing these questions would require clinically justified sampling beyond blood, for example, from ocular, neurological, joint-associated, or reproductive compartments, combined with highly optimized low-input molecular workflows, potentially including pre-extraction virion enrichment.

The host range and transmission ecology of BDBV remain incompletely understood. Experimental infection studies in domestic swine indicate that BDBV can infect pigs and produce viral shedding with evidence of intraspecies transmission [28]. These data do not establish swine as an epidemiologically important reservoir or amplifier in human outbreaks. They do, however, show that animal host studies remain relevant to filovirus surveillance and outbreak investigation.

Across the following sections, direct BDBV evidence is separated from BDBV-inclusive platform data and extrapolation from EBOV, Sudan virus (SUDV), Marburg virus (MARV), or broader filovirus studies. This distinction is essential because diagnostic, therapeutic, antibody, vaccine, and post-exposure claims answer different questions depending on whether they derive from human outbreak observations, animal challenge studies, authentic-virus assays, minigenome systems, GP-pseudotyped reporter-particle neutralization assays, binding studies, antigen inclusion, or platform plausibility.

## 2. Clinical, Pathogenesis, and Animal-Model Context for Countermeasure Interpretation

The BDBV evidence base is smaller than that of EBOV, but it is sufficient to show that BVD must not be treated as merely an EBOV analog. Ebolaviruses differ in genome sequence, antigenicity, virulence, immune evasion, host response, and animal-model behavior [16]. These differences limit direct transfer of EBOV-derived assumptions to BDBV.

Clinical presentation and outcome data remain limited but operationally important. In the 2007–2008 Uganda outbreak, hospitalized laboratory-confirmed patients frequently had non-bloody diarrhea, severe headache, asthenia, nausea or vomiting, myalgia, abdominal pain, dysphagia, and appetite loss; hemorrhage occurred in a minority of patients but was associated with fatal outcome [6]. In the 2012 Isiro outbreak, systemic, gastrointestinal, and pain-related symptoms were again frequent, bleeding was less common, and case management and clinical documentation were challenging in the Ebola Treatment Center setting [10]. These studies support two operational points: BVD requires laboratory confirmation, and clinical documentation is not a secondary issue but a prerequisite for evaluating supportive care, diagnostics, and future countermeasure use.

Immune-response data specific to BDBV further indicate that BVD pathogenesis should not be inferred from EBOV alone. This is important because fatal EBOV disease is commonly framed around systemic inflammatory dysregulation and high pro-inflammatory cytokine responses, whereas the limited human BDBV data available so far do not simply reproduce that pattern. In human samples from the 2007–2008 BDBV outbreak, fatal infection was associated with higher viral antigen levels, low concentrations of several pro-inflammatory cytokines, including interleukin-1 alpha, interleukin-1 beta, interleukin-6, and tumor necrosis factor alpha, and high concentrations of interleukin-10 [29]. Antibodies were detected even in some fatal acute infections [29]. In a BDBV rhesus macaque model, survival was associated with early activation of adaptive immunity, stronger anti-BDBV antibody responses, and reduced myeloid-derived suppressor cell-related signaling, whereas fatal disease was associated with higher viral loads and immune dysregulation [30]. These findings do not show that BDBV pathogenesis is unrelated to EBOV pathogenesis, but they do show that the available BDBV immune-response profile is not identical to the EBOV-derived fatal-disease model. BDBV-specific pathogenesis and countermeasure studies are therefore required rather than reliance on EBOV extrapolation alone.

Because prospective efficacy studies during rare and sporadic BVD outbreaks are rarely feasible, carefully justified animal models remain central to preclinical pathogenesis and countermeasure evaluation. Ferrets infected with wild-type BDBV developed lethal disease without viral adaptation, including viremia, viral shedding, rash, thrombocytopenia, lymphocyte changes, biochemical evidence of organ dysfunction, and viral antigen in multiple organs [31]. This model is relevant for pathogenesis and countermeasure screening, although results from ferrets, immunodeficient mice, surrogate-virus systems, and NHPs answer different questions and cannot be treated interchangeably.

Genomics is also central to outbreak recognition, species assignment, transmission reconstruction, and assessment of countermeasure relevance [14]. In the BDBV context, sequencing has particular importance because diagnostic assays optimized for previously more frequently encountered species, such as *Orthoebolavirus zairense* (EBOV) or *Orthoebolavirus sudanense* (SUDV), can miss divergent viruses. Genomic confirmation also enables assessment of assay target compatibility and identification of sequence features relevant to therapeutic or vaccine evaluation. For the 2007–2008 Uganda outbreak, 11 viral genome sequences were submitted to the National Center for Biotechnology Information (NCBI) Nucleotide database (as of 22 May 2026). However, these entries include only two specifically named isolates: “Butalya-811250” (GenBank accessions KR063673.1 and FJ217161.1/NC_014373.1) and “Bundibugyo-200706291” (GenBank accessions MK028856.1, MK028835.1, and KU182911.1). Several submissions therefore appear to represent different sequencing platforms, assembly approaches, or presumably different cell-culture passages rather than independent biological samples.

A similar issue applies to the 2012 DRC outbreak dataset. Initial genomic analysis confirmed the first recognized emergence of BDBV in the DRC and showed that the available 2012 genomes were approximately 98.6% identical to the 2007 Uganda BDBV genome [9]. The four initially sequenced patient samples were highly similar to each other and were submitted under GenBank accessions KC545393.1, KC545394.1, KC545395.1, and KC545396.1 [9]. In total, 23 viral genome sequences from the 2012 DRC outbreak were submitted to the NCBI Nucleotide database (as of 22 May 2026). Among the 19 subsequently sequenced viral genomes submitted in 2020, at least five isolate names (“Isiro-20120022-1”, “Isiro-20120004”, “Isiro-20120074-1”, “Isiro-20120115-1”, and “Isiro-20120130”) occur as two different GenBank accessions each. These are likely samples from the same patients, but were sequenced after different virus-isolation or cell-culture passage histories. Later retrospective sequencing expanded the 2012 dataset and challenged a simple single-introduction interpretation by identifying evidence compatible with earlier circulation and more than one spillover or introduction scenario [11].

When interpreting expanded genomic datasets from outbreak responses, careful attention to whether sequences represent independent biological samples or technical replicates is critical. Multiple sequences of the same isolate can inflate apparent genetic diversity and create spurious evidence for multiple introductions through pseudo-replication, while independently acquired culture-adaptive substitutions in shared cell-line systems can create homoplastic phylogenetic signals and artifactually cluster otherwise unrelated outbreak samples. Phylogenetic and phylogeographic inference should therefore be based on a dereplicated dataset with one representative per biological sample, reserving replicates for quality-control validation of group structure and technical-noise estimation. This evolution in interpretation illustrates why sparse genomic sampling should be treated cautiously during early outbreak reconstruction, and why expanded datasets require explicit attention to sample independence.

Clinical and countermeasure frameworks centered on EBOV remain essential but incomplete for BDBV. Broad reviews of Ebola disease summarize clinical management, infection prevention and control, therapeutics, vaccines, and survivor care, but they generally treat BDBV as one ebolavirus among several rather than as the primary subject [3]. Similarly, filovirus vaccine reviews emphasize that vaccine development has advanced fastest for EBOV and that countermeasures for other filoviruses require additional evidence [15,32].

This review therefore uses comparative filovirus literature only as a guardrail. Comparisons are included where they clarify why EBOV-focused diagnostics, vaccines, or antibodies cannot be assumed to cover BDBV; where they identify broadly reactive platforms that include BDBV experimentally; or where they define implementation questions relevant to BVD preparedness.

## 3. Molecular Diagnostics and Species Identification

The central diagnostic issue in BVD is breadth of detection. Species-specific RT-qPCR and cartridge-based assays are essential in defined outbreak contexts, but they are not sufficient as stand-alone tools when the causative filovirus species is unknown. A robust diagnostic workflow should combine broad filovirus screening, sequencing-based species assignment, and subsequent species-specific confirmation [33,34]. Table 1 summarizes nucleic acid amplification assays relevant to BDBV diagnostic recognition and outbreak response.

In the 2007–2008 BDBV outbreak, the causative virus was identified only after diagnostic escalation beyond assays targeting then-known filoviruses. The outbreak investigation identified a divergent ebolavirus that differed by more than 30% at the genome level from previously recognized ebolaviruses [4]. Evidence of acute ebolavirus infection was detected by broadly reactive antigen and serological assays [4]. By contrast, highly sensitive RT-qPCR assays specific for known Zaire and Sudan ebolaviruses and Marburg viruses were initially negative [4]. A more broadly reactive filovirus large polymerase gene (L) reverse transcription PCR (RT-PCR), followed by sequence analysis of the resulting amplicons, revealed that the virus was distinct from known ebolaviruses [4]. Subsequent genome recovery combined random-primed metagenomic pyrosequencing from patient serum with primer-walking completion from a virus isolate, illustrating how broad molecular screening and sequencing-based escalation together enabled recognition of BDBV [4]. The implication is not that the RT-qPCR assays lacked analytical sensitivity, but that the available assays lacked appropriate target breadth for a divergent virus. After BDBV had been identified by broader testing and sequencing, a BDBV-specific nucleoprotein (NP)-targeted RT-qPCR assay was developed for species-specific confirmation [4].

The May 2026 outbreak provides a contemporary operational example of this diagnostic escalation pathway. WHO reported that initial testing of 20 samples from the Rwampara Health Zone at the Provincial Public Health Laboratory in Bunia using standard “Ebola Xpert” testing was negative for EBOV [12]. In the published EBOV diagnostic literature, this cartridge-based RT-qPCR workflow corresponds to the Xpert^®^ Ebola Assay on GeneXpert^®^ Instrument Systems (Cepheid, Sunnyvale, CA, USA) [24,25]. Rapid cartridge-based EBOV assays have been analytically validated and field-tested in EBOV outbreak settings, supporting near-patient use when the target virus is appropriate [24,25]. However, such evidence does not establish pan-orthoebolavirus performance [24,25]. Samples were then referred to INRB for further analysis, where eight of 13 blood samples from the Rwampara Health Zone were confirmed as *Orthoebolavirus*-positive by RT-PCR on 15 May 2026, followed by genomic sequencing that identified the virus species as BDBV [12]. The CDC 19 May 2026 situation report similarly described initial negative EBOV testing in the DRC, followed by identification of Bundibugyo virus by genetic fingerprinting after eight of 13 samples tested positive and five were inconclusive [19]. The available public reports do not provide the full assay-by-assay analytical workflow, primer or probe targets, or genome-generation details for the 2026 confirmation sequence [12,19]. This sequence of events supports diagnostic algorithms that do not stop at an EBOV-focused negative result when clinical and epidemiological suspicion for filovirus disease remains high.

Published broad filovirus nucleic acid amplification assays relevant to BDBV recognition include several formats rather than a single pan-filovirus screening tool [35,36,37,38,39,40]. These assays differ in target gene, amplicon length, reaction format, intended use, validation material, and suitability for diagnostic versus surveillance settings. Nucleoprotein (NP)-targeting conventional RT-PCR assays include the one-step assay that detected BDBV ribonucleic acid (RNA) together with other then-known filoviruses, resulting in a 594-base-pair (bp) amplicon [37], and a one-step pan-filovirus RT-PCR screening assay generating a 317 bp sequenceable NP amplicon that detected RNA from virus isolates of BDBV, Taï Forest virus, Reston virus, SUDV, EBOV, and MARV [35].

The RealStar^®^ commercial assay family (altona Diagnostics GmbH, Hamburg, Germany) is based on an L-targeting broad screening hydrolysis probe RT-qPCR test that covered the filovirus collections of European biosafety level 4 (BSL-4) laboratories before the discovery of BDBV in 2007 [36]. Each of the three RealStar^®^ assays addresses a different aim: *Filoviridae* family-wide screening, *Orthoebolavirus* genus-level screening, and species-identification follow-up testing. The RealStar^®^ Filovirus Screen RT-PCR Kit 1.0 (altona Diagnostics GmbH, Hamburg, Germany) [41] facilitates broad filovirus detection with genus differentiation on recommended platforms [38]. This test functions as a practical reference assay at the beginning of a new filovirus outbreak, when no optimized BDBV-specific RT-qPCR has yet been established, commercially implemented, or newly listed for emergency use. It was listed under the WHO Ebola Virus Disease Emergency Use Assessment and Listing (EUAL) framework and considered eligible for WHO procurement for Ebola virus disease and Marburg virus disease emergency testing in 2014 [42]. Despite this role, WHO still lists the assay, as of 8 July 2026, only as an ongoing EUL application for BDBV nucleic-acid detection, not as a final EUL decision or procurement recommendation [43]. This assay nevertheless played an essential role in revealing the causative agent of the 2026 BVD outbreak, as it provided the first positive molecular evidence of filovirus infection in eight clinical BVD samples from the DRC and one sample from Uganda, enabling subsequent species identification by amplicon sequencing [44]. The related RealStar^®^ Ebolavirus RT-PCR Kit 1.0 (altona Diagnostics GmbH, Hamburg, Germany) [45] provides BDBV-inclusive ebolavirus screening without species differentiation and was authorized by the U.S. Food and Drug Administration (FDA) for ebolavirus RNA detection in 2014 [46]. Both RealStar^®^ assays, the Filovirus Screen Kit and the Ebolavirus Kit, were recommended by the Africa CDC on 22 May 2026 for the detection of BDBV [47].

Broad biosurveillance and discovery-oriented assays include a two-step pan-filovirus dye-based RT-qPCR assay targeting NP, which detected BDBV and other mammalian-filovirus synthetic constructs [39], and a high-throughput L-targeting dye-based RT-qPCR assay generating a 416 bp L-gene amplicon, which detected synthetic mammalian-filovirus RNA templates, including that of BDBV, with amplicon sequencing-based species identification [40].

These broad assays are useful when the causative filovirus species is uncertain or for the discovery of previously unknown virus species or strains, but they do not all answer the same diagnostic question. Conventional endpoint RT-PCR assays [35,36,37] provide amplicons long enough for species identification by Sanger sequencing. Dye-based RT-qPCR assays [39,40] are useful for surveillance but require confirmatory analysis by high-resolution melt curve analysis, amplicon sequencing, or other follow-up methods. Species-specific panel assays require selective inclusion of their BDBV-specific component [48,49]. For example, one of the four master mixes of the Research Use Only (RUO) RealStar^®^ Filovirus Type RT-PCR Kit 2.0 (altona Diagnostics GmbH, Hamburg, Germany) consists of a combined SUDV/BDBV assay with two distinct fluorophore channels, while the other three master mixes represent a duplex Reston virus (RESTV)/Taï Forest virus (TAFV), an EBOV, and a MARV RT-qPCR test [50,51,52].

By contrast, previously developed cartridge-based EBOV-specific assays occupy a narrower but important diagnostic niche. Their main value is rapid, standardized near-patient testing when EBOV is the relevant target, whereas their limitation is target scope rather than cartridge-based technology itself [24,25]. In a BDBV-compatible clinical or epidemiological context, an EBOV-negative cartridge result should be interpreted within a tiered algorithm that includes broad filovirus RT-PCR or RT-qPCR and sequencing-based species assignment.

Because the 2026 BVD outbreak is evolving rapidly, Table 1 was expanded to include recent commercial molecular platforms documented in WHO, regulatory, manufacturer, or outbreak-response sources, even where peer-reviewed BDBV-specific data remain limited. The following assays represent commercial near-point-of-care (PoC) closed systems that integrate sample processing and detection by RT-qPCR. The BioFire^®^ Global Fever Special Pathogens Panel (BioFire Defense, LLC, Salt Lake City, UT, USA) is a multiplex syndromic, two-stage nested, dye-based RT-qPCR assay for detection of 16 fever-causing pathogens, including BDBV and other ebolavirus species [53,54]. The Xpert^®^ Hemorrhagic Fever Panel (Cepheid, Sunnyvale, CA, USA) detects the ebolaviruses EBOV, SUDV, TAFV, and BDBV, although without species differentiation, as well as the other viral hemorrhagic fever (VHF) agents MARV, Lassa virus, and Crimean–Congo hemorrhagic fever virus (CCHFV) [55,56]. The RADIONE Ebola Detection Kit RP017 (KH Medical Co., Ltd., Pyeongtaek-si, Republic of Korea) [57], already employed during the current 2026 outbreak, was used to test 148 suspected BVD blood samples from the Ituri Province, DRC, and showed 95% overall agreement with the RealStar® Filovirus Screen RT-PCR Kit 1.0 [58]. While already widely employed in the 2026 outbreak in the DRC and recommended for BDBV detection by the Africa CDC [47], the WHO still lists the assay, as of 8 July 2026, only as an ongoing EUL application for BDBV nucleic-acid detection, not as a final EUL decision or procurement recommendation [43]. The same WHO EUL application status applies to the RADIONE Pan-Ebola Genotyping & Marburg Multiplex Kit (RP038, KH Medical Co., Ltd., Pyeongtaek-si, Republic of Korea) [59], launched in late May 2026, which the manufacturer describes as detecting MARV and the *Orthoebolavirus* species BDBV, EBOV, SUDV, TAFV, and RESTV, with species-level differentiation reported for MARV, BDBV, EBOV, and SUDV.

The WHO listed the Liferiver™ Ebola Virus (EBOV) Real Time RT-PCR Kit (QR-0220-02, Shanghai ZJ Bio-Tech Co., Ltd., Shanghai, China) [60] as the first BDBV nucleic-acid test under its Emergency Use Listing (EUL) procedure on 2 July 2026 [61], although the current public assessment report was still pending as of 8 July 2026 [62]. This is the commercial RT-qPCR test previously considered eligible for WHO procurement under the 2015 Ebola EUAL framework [63]. The manufacturer’s Instructions for Use (IFU) report analytical sensitivity using “EBOV pseudovirus plasmids” diluted across several concentrations [60]. Testing was performed with three kit lots and three repeats per concentration, followed by 20 confirmatory repeats at the lowest positive concentration [60]. The IFU concludes a minimum detection level of 1 × 10^3^ copies/mL for all four ebolavirus plasmid materials, but does not report a probit analysis, confidence interval, dilution-series table, standard curve, or biological-matrix effect [60]. With the specified 5 µL template input in a 25 µL reaction, 1 × 10^3^ copies/mL corresponds nominally to approximately five copies per reaction. By contrast, the 2019 WHO public report stated that this did not mean WHO prequalification and described a limited independent laboratory evaluation using infectious EBOV Makona cell-culture supernatant spiked into healthy-donor whole blood, with a 95% limit of detection of 23.9 RNA copies/reaction and a wide 95% confidence interval of 13.4–405.9 RNA copies/reaction [63]. The assay thermoprofile is also notable, combining a short 45 °C 10 min reverse-transcription step with a prolonged 95 °C 15 min activation/denaturation step before 40 cycles of 95 °C 15 s and 60 °C 60 s [60]. This profile may be compatible with an older chemically activated hot-start polymerase formulation, but it makes transparent validation with RNA-containing material and clinical matrices particularly important. For BDBV, the IFU states that primers and probes cover six listed historical BDBV accessions, but they only represent different submissions of the same genome of a BDBV isolate from the 2007–2008 outbreak in Uganda (GenBank IDs: FJ217161, NC_014373, and JA489018) and the genomic sequences of three BDBV isolates from the 2012 outbreak in the DRC (GenBank IDs: KC545393, KC545394, and KC545395). As with the other commercial molecular platforms discussed above, the Liferiver IFU does not disclose primer/probe sequences, the target gene or region, amplicon length, or target-region checking against 2026 BVD outbreak genomes [60]. This underscores the need for timely peer-reviewed analytical and clinical BDBV validation and publication of Minimum Information for Publication of Quantitative Real-Time PCR Experiments (MIQE)-level assessment details.

**Table 1:** Nucleic acid amplification assays relevant to Bundibugyo virus diagnostic recognition and outbreak response. Entries are ordered by year of publication. Abbreviations: BDBV, Bundibugyo virus; bp, base pairs; BSL-4, biosafety level 4; BVD, Bundibugyo virus disease; CCHFV, Crimean–Congo hemorrhagic fever virus; DRC, Democratic Republic of the Congo; EBOV, Ebola virus; EUL, Emergency Use Listing; GP, glycoprotein gene; L, large polymerase gene; LoD95, 95% limit of detection; MARV, Marburg virus; MGB, minor groove binder; NP, nucleoprotein gene; PCR, polymerase chain reaction; RESTV, Reston virus; RNA, ribonucleic acid; RT-LAMP, reverse transcription loop-mediated isothermal amplification; RT-PCR, conventional endpoint reverse transcription polymerase chain reaction; RT-qPCR, real-time reverse transcription quantitative polymerase chain reaction; RUO, Research Use Only; SUDV, Sudan virus; TAFV, Taï Forest virus; VHF, viral hemorrhagic fever. Minimum Information for Publication of Quantitative Real-Time PCR Experiments (MIQE) terminology is used [64].

Year	Assay or Publication	Format	Target	Diagnostic Role	BDBV Status	Main BDBV-Related Limitation	Source
2007	Diagnostic RT-qPCR kit for filoviruses based on European BSL-4 strain collections	Hydrolysis probe RT-qPCR	L	Pre-BDBV broad filovirus screen	No BDBV tested. Relevant as L-gene assay lineage for later BDBV-inclusive RealStar assay.	Original paper does not support a direct BDBV detection claim.	[36]
2008	BDBV-specific NP RT-qPCR developed during the 2007–2008 Uganda outbreak	Hydrolysis probe RT-qPCR	NP	Species-specific BDBV confirmation after genome-informed assay design	Primer/probe sequences published; assay evaluated on initial outbreak samples.	Not suitable as the first-line assay when the causative filovirus species is unknown.	[4]
2010	Comprehensive hemorrhagic fever virus PCR panel	Hydrolysis probe RT-qPCR panel, including MGB assays	BDBV NP, 74 bp	Species-specific differential panel	BDBV-specific NP assay included.	Not a pan-filovirus assay. The BDBV component must be run.	[48]
2011	NP RT-PCR for detection of all known filovirus species	One-step conventional RT-PCR	NP, 594 bp	Broad endpoint filovirus screen	BDBV RNA detected.	Requires product confirmation and sequencing for species assignment.	[37]
2014	RealStar^®^ Ebolavirus RT-PCR Kit 1.0 (altona Diagnostics GmbH, Hamburg, Germany)	Commercial hydrolysis probe RT-qPCR	L	*Orthoebolavirus* species screening: EBOV, SUDV, TAFV, RESTV, BDBV	BDBV in vitro-transcribed RNA tested.	No *Orthoebolavirus* species differentiation.	[45]
2015/2016	Xpert^®^ Ebola Assay/GeneXpert^®^ (Cepheid AB, Solna, Sweden)	Commercial cartridge-based one-step hydrolysis probe RT-qPCR	EBOV GP and NP	EBOV-focused near-patient assay	Operational negative comparator in the 2026 BDBV investigation.	Not a BDBV assay and not a pan-orthoebolavirus assay.	[12,19,24,25]
2016	RealStar^®^ Filovirus Screen RT-PCR Kit 1.0 (altona Diagnostics GmbH, Hamburg, Germany)	Commercial hydrolysis probe RT-qPCR	L	BDBV-inclusive broad filovirus screen with genus differentiation	BDBV in vitro transcript tested. LoD95 was 1.8 RNA copies/µL eluate on recommended platforms. First test to detect viral RNA in clinical BVD samples of the 2026 outbreak (8 in DRC, 1 in Uganda).	Modified from the 2007 L-gene assay. Exact kit oligonucleotides were not disclosed. No direct BDBV benchmark against the original assay [36] was reported.	[38,41,44]
2019	Pan-filovirus one-step RT-PCR screening assay	One-step conventional RT-PCR	NP, 317 bp	Pan-filovirus screening with sequenceable amplicon	RNA extracted from BDBV isolate detected.	Screening and discovery assay. It is not a replacement for optimized RT-qPCR.	[35]
2019	Four-species ebolavirus RT-LAMP chip	RT-LAMP	GP	Species-specific RT-LAMP platform	BDBV channel included.	Platform and synthetic-target evidence. Not pan-filovirus.	[49]
2021	RealStar^®^ Filovirus Type RT-PCR Kit 2.0 (altona Diagnostics GmbH, Hamburg, Germany)	Commercial hydrolysis probe RT-qPCR	L	Suite of four different assays for species-specific detection of MARV, EBOV, RESTV/TAFV, SUDV/BDBV	BDBV channel of SUDV & BDBV assay; BDBV synthetic RNA template tested.	Currently holds Research Use Only (RUO) status; head-to-head validation testing of clinical samples from the 2026 BVD outbreak in comparison with RealStar^®^ Filovirus Screen RT-PCR Kit 1.0 ongoing.	[50,51,52]
2023	Pan-filovirus RT-qPCR assay for bat biosurveillance	Two-step dye-based RT-qPCR	NP, 157 bp	Mammalian filovirus biosurveillance	BDBV synthetic NP construct tested.	Biosurveillance assay. Positive results require confirmation.	[39]
2023	BioFire^®^ Global Fever Special Pathogens Panel	Commercial two-stage, multiplexed, nested, dye-based RT-qPCR assay	Proprietary target	Screening panel for 16 fever-causing pathogens, *Orthoebolavirus* species: BDBV, EBOV, SUDV, TAFV, RESTV	RNA extracted from BDBV isolate detected.	Closed system output: “*Ebolavirus* spp. *Detected*”; BDBV species confirmation required.	[53]
2024	High-throughput polymerase-targeted RT-PCR	Two-step dye-based RT-qPCR plus amplicon sequencing	L, 416 bp	Broad mammalian filovirus screening and discovery	BDBV synthetic RNA template tested.	BDBV was not included in the inactivated-isolate panel. Species assignment requires confirmation or sequencing.	[40]
2026	RADIONE Ebola Detection Kit (RP017, KH Medical Co., Ltd., Pyeongtaek-si, Republic of Korea)	Commercial cartridge-based one-step hydrolysis probe RT-qPCR	Proprietary target	Detects *Orthoebolavirus* species: BDBV, EBOV, SUDV, TAFV, RESTV	BDBV detected in patient samples in 2026 DRC outbreak.	No *Orthoebolavirus* species differentiation.	[57,58]
2026	RADIONE Pan-Ebola Genotyping & Marburg Multiplex Kit (RP038, KH Medical Co., Ltd., Pyeongtaek-si, Republic of Korea)	Commercial cartridge-based one-step hydrolysis probe RT-qPCR	Proprietary target	Detects MARV and *Orthoebolavirus* species: BDBV, EBOV, SUDV, TAFV, RESTV; differentiates MARV, BDBV, EBOV, SUDV	Detection of what type of BDBV synthetic construct not disclosed in detail.	Unclear status on detection beyond BDBV synthetic construct.	[59]
2026	Xpert^®^ Hemorrhagic Fever Test	Commercial cartridge-based one-step hydrolysis probe RT-qPCR	Proprietary target	VHF panel: MARV, CCHFV, Lassa virus, and *Orthoebolavirus* species: BDBV, EBOV, SUDV, TAFV	Validated with live BDBV-contrived human whole blood.	No *Orthoebolavirus* species differentiation.	[55]
2026	Liferiver™ Ebola Virus (EBOV) Real Time RT-PCR Kit (Shanghai ZJ Bio-Tech Co., Ltd., Shanghai, China)	Commercial one-step hydrolysis probe RT-qPCR	Proprietary target	WHO EUL-listed molecular test for BDBV 2026 outbreak response testing; *Orthoebolavirus* species: BDBV, EBOV, SUDV, TAFV	Public report lists six historical BDBV sequences covered by primers/probes.	Pending WHO EUL report on independent viral RNA validation; BDBV analytical limit of detection supported only by manufacturer’s synthetic plasmid-target data.	[60,61,62]

Sequencing complements nucleic acid amplification testing and occupies several distinct positions in this diagnostic landscape; sequencing-based approaches are summarized in Table 2. Sanger sequencing of sufficiently long amplicons can provide species assignment after broad RT-PCR [4,35,37]. Whole-genome sequencing of BDBV can support lineage assignment, transmission reconstruction, and monitoring of diagnostic targets [4,9,11,14]. Agnostic or semi-agnostic metagenomic sequencing can be particularly valuable when targeted assays are negative or when the clinical syndrome suggests a high-consequence viral infection but the causative agent is uncertain [14]. Field-deployable sequencing, including Oxford Nanopore Technologies plc, Oxford, UK (ONT)-based approaches, has demonstrated utility for near-real-time EBOV genomic surveillance and can complement targeted diagnostics by enabling rapid genomic characterization in outbreak settings [65]. In the BVD outbreak context, sequencing should therefore be used within an informed and flexible strategy that accounts for the outbreak phase, diagnostic question, sample quality, viral load, biosafety requirements, available infrastructure, and need for rapid species assignment or broader genomic epidemiological characterization, rather than as a replacement for validated BDBV molecular assays optimized for reliable and scalable outbreak testing [66].

The diagnostic conclusion is therefore a layered model: broad screening when species identity is uncertain, sequencing for confirmation and optimized species-specific RT-qPCR once the target is known as well as lineage assignment, outbreak reconstruction, and genomic surveillance. The May 2026 outbreak, the third recognized BVD outbreak to date, again demonstrated why BVD diagnostic workflows must accommodate species-level uncertainty [12,19]. Africa CDC’s May 2026 response similarly highlighted laboratory coordination, digital surveillance and data management, cross-border preparedness, and assessment of medical countermeasure appropriateness after sequencing confirmed the ebolavirus species [21].

In remote, infrastructure-limited, or insecure outbreak settings, including forested or mining-associated communities where sample transport to molecular laboratories may be delayed, BDBV-compatible antigen rapid diagnostic tests (RDTs) could substantially improve rapid triage, isolation decisions, and the proportion of suspected cases receiving at least presumptive testing. However, no antigen RDT is currently recommended, listed, approved, or clinically validated for BDBV diagnosis in the 2026 outbreak. Africa CDC’s Diagnostics Advisory Committee concluded that no antigen RDT currently meets the required specifications for recommendation [67], while WHO’s BDBV EUL pathway currently addresses only nucleic-acid detection tests [68]. The Day 45 International Pandemic Preparedness Secretariat (IPPS) update reported that 27 antigen RDTs had been identified, 22 reviewed, and 11 selected for evaluation, including planned field evaluation in the DRC and Uganda and complementary BSL-4 analytical testing [67]. Existing Ebola antigen RDT evidence, however, is largely EBOV-derived. The OraQuick^®^ Ebola Rapid Antigen Test (OraSure Technologies, Inc., Bethlehem, PA, USA) is an FDA de novo-authorized lateral-flow immunoassay for presumptive detection of antigens from viruses within the genus *Orthoebolavirus*, does not provide species differentiation, and requires confirmatory testing [69]. The FDA documentation reports analytical reactivity with SUDV and BDBV, in addition to EBOV, but not with tested TAFV or RESTV materials under the reported conditions [69]. Clinical performance data remain mainly EBOV-based: OraQuick^®^ Ebola RDT showed 84.0% positive percent agreement in archived Ebola virus disease venous whole-blood samples [69,70], while a head-to-head evaluation in eastern DRC EBOV outbreak samples reported 61.6% sensitivity and 98.1% specificity versus the Xpert^®^ Ebola RT-qPCR (GeneXpert^®^) (Cepheid AB, Solna, Sweden) assay [71]. BDBV antigen RDTs should therefore be treated as an urgent preparedness gap, not as replacements for validated RT-qPCR, broad molecular testing, or sequencing-based species assignment.

The WHO Filovirus Research and Development Roadmap emphasizes the need to strengthen diagnostic and field laboratory capacity, develop and validate field-suitable serological and PCR tests, support antigen rapid diagnostic test development, expand multiplex PCR and sequencing approaches, harmonize assays across countries, and strengthen reagent repositories and evaluation standards [72].

## 4. Therapeutic Approaches

### 4.1. Small-Molecule Antivirals

Direct antiviral evidence for BDBV remains limited. A newly developed BDBV minigenome system enabled comparison of BDBV and EBOV polymerase-complex activity and provided a platform for antiviral susceptibility testing [73]. The same study also tested remdesivir against infectious BDBV and EBOV in HepG2 cells, reporting 90% effective concentration values of 109.6 nM for BDBV and 284.1 nM for EBOV based on infectious-virus readout [73]. Thus, the study provides BDBV-specific remdesivir susceptibility evidence at two in vitro levels: minigenome polymerase activity and authentic-virus cell-culture inhibition, but does not establish BDBV animal protection, post-exposure efficacy, or clinical benefit [73].

Remdesivir has stronger evidence in EBOV models, including NHP efficacy, but those data should not be treated as BDBV efficacy. In EBOV models, GS-5734/remdesivir showed therapeutic efficacy in rhesus monkeys, supporting remdesivir as a filovirus antiviral candidate [74]. For BDBV, the appropriate interpretation is narrower: remdesivir is a plausible BDBV-relevant antiviral candidate supported by a BDBV minigenome system, authentic-virus cell-culture inhibition, and broader filovirus precedent, not a validated BVD therapy.

Polymerase-sequence variation further complicates remdesivir interpretation. Polymerase substitutions associated with reduced remdesivir susceptibility have been described for EBOV [75]. At the homologous position corresponding to EBOV L polymerase residue 562, BDBV naturally encodes alanine, equivalent to the T562A substitution observed after remdesivir selection in EBOV [75]. Whether this substitution functionally reduces remdesivir susceptibility in BDBV polymerase has not been directly established. Current BDBV data support polymerase susceptibility, but natural polymerase diversity warrants validation with diverse BDBV isolates under clinically relevant pharmacokinetic assumptions. The currently available evidence does not establish clinical efficacy, post-exposure prophylaxis efficacy, or clinically meaningful resistance for BDBV.

Obeldesivir is relevant because oral administration could be operationally advantageous for post-exposure or early treatment strategies, particularly compared with remdesivir, which requires intravenous administration. Post-exposure protection has been reported in NHP models of SUDV, MARV, and EBOV infection [76,77,78]. These studies support obeldesivir as a broad filovirus antiviral candidate, but they do not establish BDBV efficacy. No direct BDBV obeldesivir efficacy data were identified in the sources reviewed here.

Galidesivir, formerly developed as BCX4430 by BioCryst Pharmaceuticals, Inc. (Durham, NC, USA) and now advanced by Island Pharmaceuticals Ltd. (Hawthorn East, VIC, Australia), is another nucleoside analog relevant to the 2026 outbreak. Ugandan regulatory and ethics authorities have granted approval for compassionate use under a WHO-recognized Monitored Emergency Use of Unregistered and Investigational Interventions (MEURI) protocol during the current BVD outbreak [79,80]. The program is described as prospectively collecting clinical, safety, and virological data in infected patients, but this should not be interpreted as regulatory approval of galidesivir or as evidence of clinical efficacy. Galidesivir has filovirus-supporting preclinical evidence, including activity against EBOV and MARV in cell-culture and NHP models, and an EBOV rhesus macaque study reported protection with loading-dose regimens initiated two or three days after challenge [81,82]. No direct BDBV efficacy data were identified in the sources reviewed here. Galidesivir should therefore be interpreted as a broad-spectrum filovirus antiviral candidate with potential to generate rare human BVD outbreak data, not as an established BDBV therapy.

Favipiravir has been evaluated in filovirus animal models, but available evidence does not establish BDBV efficacy [83]. No direct BDBV efficacy evidence was identified for favipiravir, and no head-to-head comparison of remdesivir, obeldesivir, favipiravir, or other small-molecule antiviral candidates in authentic BDBV infection models was identified in the sources reviewed here.

None of the small-molecule antivirals discussed here is approved for BDBV infection or BVD [23]; regulatory availability is indication-specific, with intravenously administered remdesivir approved or authorized for coronavirus disease 2019 (COVID-19) by the FDA [84] and the European Medicines Agency (EMA) [85], favipiravir lacking approval by these agencies but authorized in selected countries for restricted indications such as novel or re-emerging influenza in Japan [86] or restricted emergency use for mild-to-moderate COVID-19 in India [87], and obeldesivir remaining investigational and not approved by any regulatory authority [88].

Recent EBOV NHP data comparing the small-molecule compound remdesivir and the antibodies monoclonal antibody 114 (mAb114), REGN-EB3/INMAZEB (atoltivimab/maftivimab/odesivimab-ebgn), and ZMapp under uniform late-treatment conditions showed incomplete protection for all single interventions and support continued evaluation of combination approaches for advanced filovirus disease [89]. These findings are useful for framing treatment timing and combination-therapy rationale, but they are EBOV-specific and should not be interpreted as BDBV efficacy evidence.

The WHO Filovirus Research and Development Roadmap supports the same research direction at the preparedness level: therapeutics should be developed and evaluated for under-researched filoviruses, including monoclonal antibodies and antiviral agents across the family, host-directed therapies, interventions targeting viral persistence and sanctuary sites, and harmonized clinical evidence generation through pre-approved core protocols and outbreak-ready platforms [72]. These roadmap priorities support BDBV trial readiness and research prioritization, not claims of efficacy for any specific BDBV therapeutic candidate. The small-molecule antiviral therapeutics evidence relevant to BDBV is summarized in Table 3 according to candidate class, evidence category, BDBV-specific findings, and main caveat.

### 4.2. Antibody, Protein, Peptide, and Nucleic Acid Therapeutics

This therapeutic class covers antibody-, protein-, peptide-, and nucleic-acid-based candidates, including monoclonal antibodies, antibody cocktails, bispecific antibodies, messenger ribonucleic acid (mRNA)-encoded antibodies, nanobodies, peptides, and epitope-focused design approaches. It is used here as a pharmacological grouping of non-vaccine therapeutics distinct from small-molecule antivirals and vaccines, not as a regulatory classification. The strength of BDBV-relevant evidence varies substantially.

Monoclonal antibodies derived from BDBV survivors provide direct evidence that natural BDBV infection can elicit cross-reactive humoral responses. Survivor antibody studies show that natural ebolavirus infection can elicit cross-reactive and neutralizing antibodies, including antibodies derived from BDBV survivors [90]. Monoclonal antibody BDBV223 is a key example. Identified from a BVD survivor, BDBV223 neutralizes BDBV and EBOV but not SUDV, illustrating both the feasibility and limits of cross-reactive antibody recognition [91]. It should be described as cross-reactive across two ebolaviruses, not as pan-orthoebolavirus. Mechanistically, BDBV223 also has evidence beyond simple neutralization. EBOV and BDBV can spread to neighboring cells through intercellular connections in a process dependent on actin and T-cell immunoglobulin and mucin domain 1 (TIM-1) [92]. The membrane-proximal external region (MPER)-specific antibody BDBV223 inhibits BDBV spread through intercellular connections in a bone marrow stromal antigen 2 (BST2/tetherin)-dependent manner [92]. This finding strengthens BDBV223 as a mechanistically informative antibody, but it remains in vitro mechanistic evidence and should not be interpreted as animal or clinical efficacy.

Monoclonal antibody BDBV289-N provides direct BDBV NHP therapeutic evidence, but its survival result requires careful interpretation. In rhesus macaques challenged with BDBV, recombinant BDBV289-N, a glycan-cap-specific antibody derived from BDBV survivor antibody work, was associated with survival of all six treated animals when treatment was initiated as late as eight days after virus challenge [93]. However, six of ten untreated controls in the same study also survived [93]. This study is therefore important direct BDBV antibody evidence, but survival efficacy should not be overstated; the result is best interpreted as promising preclinical evidence requiring confirmation in models with more uniformly lethal challenge conditions and clinically realistic treatment windows.

Antibody model systems specific to BDBV also matter because direct BDBV testing is constrained by biosafety requirements and limited animal models. A chimeric EBOV/BDBV-GP virus caused 100% lethal infection in signal transducer and activator of transcription 1 (STAT1) knockout mice and was used to screen BDBV GP-specific antibodies [94]. In that model, BDBV223 protected three of five treated mice when administered 24 h after challenge [94]. Lethal vesicular stomatitis virus (VSV)-based surrogate mouse models for SUDV and BDBV have also been developed to enable antibody evaluation under biosafety level 2 (BSL-2) conditions [95]. Such models support in vivo screening but should not be conflated with authentic BDBV NHP efficacy.

Pan-ebolavirus antibody cocktails provide a breadth-oriented approach, but BDBV-specific claims require careful separation by assay type and model. The two-antibody cocktail MBP134AF provides broad preclinical filovirus protection in ferrets and NHPs, including direct BDBV data [96]. In the BDBV ferret model, MBP134AF protected animals against otherwise lethal challenge [96]. In the BDBV cynomolgus macaque study, five of six treated animals survived, whereas none of three phosphate-buffered saline controls survived [96]. These data make MBP134AF one of the strongest BDBV-relevant antibody-cocktail candidates identified here, while still remaining preclinical and not establishing human BVD efficacy.

Atoltivimab/maftivimab/odesivimab-ebgn (INMAZEB^®^/REGN-EB3) cocktail requires separate interpretation from its maftivimab/REGN3479 component. INMAZEB^®^ is approved for treatment of infection caused by EBOV, but this indication and the clinical efficacy evidence from the “Pamoja Tulinde Maisha” (PALM; “Together Save Lives” in Kiswahili) trial do not establish efficacy against BDBV [97,98,99]. The only direct BDBV-relevant evidence for maftivimab is component-specific in vitro evidence: maftivimab targets a conserved glycoprotein subunit 2 (GP2) fusion-loop/quaternary epitope and neutralized authentic BDBV in vitro [99]. This finding should not be interpreted as BDBV efficacy evidence. The same study showed rapid selection of resistant virus under maftivimab monotherapy in an EBOV GP-based escape system, whereas complete resistance to the INMAZEB^®^ cocktail was not observed after ten passages [99]. Moreover, public WHO prioritization material from the 2022 SUDV outbreak reported that only maftivimab among the three INMAZEB^®^ antibodies could potentially neutralize SUDV, yet preliminary NHP results showed no or limited efficacy for both the full INMAZEB^®^ cocktail and maftivimab alone [100]. Because maftivimab neutralized SUDV more potently than BDBV in the Rayaprolu et al. assay [99], these preliminary SUDV NHP data [100] substantially limit confidence that maftivimab would protect against BDBV in vivo. Maftivimab should therefore be considered an operationally discussed antibody component with authentic-virus BDBV in vitro activity [99] and regulatory [98] adjacency, but with substantially weaker BDBV efficacy evidence than BDBV289-N or MBP134AF [93,96,99,100]. MBP134AF remains the strongest BDBV-relevant antibody candidate identified here because it combines direct BDBV animal protection with a true antibody-cocktail format targeting conserved, non-overlapping GP epitopes [96], a design feature associated with increased resistance to antibody-driven viral escape [99].

Additional broadly reactive antibody studies, including multifunctional pan-ebolavirus antibodies and two-antibody protective therapy approaches, support the feasibility of targeting conserved GP sites [101,102]. Their relevance to BDBV depends on the exact assay, species, and challenge model used [101,102].

The human survivor-derived antibodies 1C3 and 1C11 recognize quaternary GP epitopes, do not cross-react with soluble GP, and were evaluated as a broad ebolavirus antibody pair [103]. BDBV relevance is strongest for 1C11 and the 1C3/1C11 combination in the EBOV/BDBV-GP chimeric STAT1 knockout mouse model [103]. In that model, 1C11 alone conferred 80% protection and the 1C3/1C11 cocktail conferred 100% protection when administered 24 h after challenge, whereas 1C3 alone did not protect [103]. The same antibody combination protected NHPs against EBOV and SUDV, but not against authentic BDBV challenge [103]. The 1C3/1C11 data therefore support broad antibody-design relevance and BDBV-GP surrogate small-animal activity, but do not provide direct authentic BDBV NHP efficacy evidence.

Other antibody approaches, including FVM04/CA45, contribute to the broader pan-ebolavirus immunotherapy landscape. Macaque monoclonal antibodies targeting conserved filovirus GP epitopes have shown pan-ebolavirus and pan-filovirus binding profiles and mouse protection evidence, but without direct BDBV challenge protection [104]. A subsequent antibody-combination study showed post-exposure protection of NHPs against EBOV and SUDV using FVM04/CA45 [105]. Protection against MARV required addition of the anti-MARV antibody MR191 [105]. These studies support broad filovirus immunotherapy design; however, direct BDBV efficacy cannot be inferred unless BDBV-specific testing is included.

Bispecific antibodies are a recent strategy to broaden coverage and reduce escape. Bispecific and cocktail-based antibody studies support dual-epitope targeting and broader ebolavirus coverage, including protection in EBOV and SUDV models and broad activity against multiple ebolavirus species [106,107,108]. Bispecific antibodies derived from the broadly neutralizing human monoclonal antibody rEBOV-515 and the human survivor-derived antibody 1C3 were engineered in CrossMab, dual-variable-domain immunoglobulin G, and immunoglobulin G-single-chain variable fragment formats and evaluated against EBOV, SUDV, and BDBV systems [109]. The immunoglobulin G-single-chain variable fragment construct S1+5 showed markedly improved neutralization against recombinant vesicular stomatitis virus (rVSV) expressing BDBV GP compared with parental antibodies and maintained neutralizing activity during serial passaging [109]. Several constructs protected type I interferon receptor-deficient (IFNAR−/−) mice in rVSV-BDBV challenge experiments [109]. This study should be included as BDBV-relevant surrogate-virus small-animal bispecific antibody evidence. It should not be interpreted as authentic BDBV NHP protection, and the authors themselves noted the need for further authentic SUDV and BDBV challenge studies under BSL-4 conditions [109].

Antibodies encoded by mRNA provide another emerging modality. An mRNA-encoded antibody approach has been described for the pan-orthoebolavirus neutralizing antibody 2G1 [110]. This platform is relevant to BDBV preparedness because mRNA delivery could accelerate antibody deployment, but the evidence remains preclinical.

Computational antibody optimization is beginning to enter the filovirus countermeasure research. A computational-experimental pipeline was used to optimize the broad antibodies ADI-15878 and ADI-15946 using a luciferase-expressing, envelope-defective lentiviral reporter-particle system generated from pNL4.3-Luc-E−R− and pseudotyped with GPs encoded by EBOV, BDBV, or SUDV GP plasmids [111]. Thus, the BDBV-relevant result reflects neutralization of lentiviral reporter particles bearing BDBV GP, not authentic BDBV. For ADI-15878, the W32G light-chain (W32G-LC) variant improved neutralization of reporter particles bearing EBOV, BDBV, or SUDV GP compared with the parental antibody [111]. For ADI-15946, optimized light-chain variants improved neutralization of reporter particles bearing SUDV GP while retaining activity against particles bearing EBOV or BDBV GP [111]. The authors performed these assays under biosafety level 2 (BSL-2) conditions, consistent with an entry/neutralization reporter-particle assay rather than authentic ebolavirus infection [111]. This is relevant as antibody-engineering evidence, but it remains GP-pseudotyped lentiviral reporter-particle and binding-stage evidence and should not be treated as authentic BDBV neutralization or protection.

Nanobody approaches have become increasingly relevant. The synthetic-library nanobody BDBV-Nb02 targets the BDBV glycan cap [112]. Neutralization was assessed using a pNL4.3-Luc-E−R− luciferase-expressing, envelope-defective lentiviral reporter-particle system pseudotyped with EBOV, BDBV, or SUDV GP; thus, the BDBV-relevant result reflects inhibition of lentiviral reporter particles bearing BDBV GP, not authentic BDBV [112]. BDBV-Nb02 neutralized particles bearing BDBV or EBOV GP, and bivalent formatting improved activity, but it did not neutralize particles bearing SUDV GP [112]. Authentic-virus and animal validation therefore remain necessary, and BDBV-Nb02 should be considered a rapid-discovery, GP-pseudotyped reporter-particle-stage BDBV candidate rather than a validated therapeutic.

The strongest nanobody-based BDBV-relevant protection data identified in this review come from camelid-derived nanobodies 1A10 and BA2 [113]. These nanobodies neutralized EBOV, SUDV, and BDBV in vitro [113]. The engineered BA2-1A10 bispecific antibody protected rodent models, including a VSV-BDBV challenge model [113]. This study raises the evidence level for nanobody/bispecific approaches from GP-pseudotyped reporter-particle-only evidence to small-animal protection in a BDBV-relevant surrogate model. It remains preclinical, and VSV-BDBV rodent protection should not be treated as equivalent to authentic BDBV NHP protection or human efficacy.

Peptide- and epitope-focused approaches may contribute to future BDBV therapeutic and vaccine design, particularly where conserved GP regions are targeted. Antibodies targeting conserved heptad repeat 2-membrane-proximal external region sites or other conserved GP regions inform rational design of broadly reactive interventions [114,115,116]. The BDBV heptad repeat 2-membrane-proximal external region epitope recognized by BDBV223 was specifically used for epitope-focused immunogen design [117]. The study identified a sterile alpha motif (SAM) domain scaffold displaying the BDBV heptad repeat 2-membrane-proximal external region epitope and tested nanoparticle-displayed immunogens in rabbits [117]. The immunogens induced robust BDBV and EBOV GP-binding antibodies and more moderate SUDV GP-binding responses, but sera did not show meaningful neutralization of authentic BDBV or VSV expressing BDBV GP except for limited activity in one peptide-immunized animal [117]. This study is valuable because it demonstrates both the promise and the difficulty of BDBV epitope-focused vaccine design: binding breadth can be induced, but neutralizing activity and protective efficacy remain unresolved.

The antibody-, protein-, peptide-, and nucleic-acid-based therapeutics evidence relevant to BDBV is summarized in Table 4 according to candidate class, evidence category, BDBV-specific findings, and main caveat. Maftivimab/REGN3479 was not ranked as a BDBV efficacy candidate in this table because its direct BDBV evidence is limited to in vitro neutralization, while public WHO SUDV prioritization material reported no or limited preliminary NHP efficacy for both the INMAZEB^®^ cocktail and the single-component maftivimab despite stronger SUDV than BDBV in vitro neutralization [99,100].

## 5. Vaccines and Post-Exposure Prophylaxis

Vaccines currently provide the most species-specific direct BDBV animal protection data. Recombinant vesicular stomatitis virus (rVSV)-based vaccines are central because they have shown rapid protection in filovirus NHP models and because licensed EBOV vaccination uses this general platform. For BDBV, however, antigen specificity remains decisive, and small animal numbers limit the strength of available preclinical efficacy estimates.

The studies on rVSV-based vaccines provide the strongest direct BDBV vaccine evidence, but their evidentiary weight differs by antigen and schedule. The most direct species-specific evidence comes from recombinant vesicular stomatitis virus expressing BDBV glycoprotein (rVSV-BDBV-GP), which protected NHPs against BDBV challenge [118]. Heterologous rVSV evidence is less direct: a short prime-boost regimen using rVSV expressing SUDV GP (rVSV-SUDV-GP) and rVSV expressing EBOV GP (rVSV-EBOV-GP) protected macaques against BDBV challenge, whereas a single blended heterologous vaccination strategy failed [118]. Single-dose monovalent heterologous rVSV-EBOV-GP vaccination also provided BDBV challenge evidence, but this should be interpreted as antigen-dependent heterologous protection rather than as a general rVSV-platform guarantee [119]. Published BDBV vaccine studies generally use small NHP cohorts and should be interpreted as preclinical feasibility rather than licensure-level evidence. WHO-convened experts also reviewed the potential role of the licensed EBOV vaccine ERVEBO. They concluded that ERVEBO is not licensed for prevention of BVD and that evidence for cross-protection against other ebolavirus species remains limited and inconclusive; WHO therefore recommended that ERVEBO should not be used outside carefully designed research settings during BVD outbreaks [23].

Pan-filovirus vaccine platforms are relevant where BDBV antigens or BDBV challenge data are explicit. A highly attenuated quadrivalent VesiculoVax recombinant vesicular stomatitis virus-based filovirus vaccine formulation (rVSV-Filo) contained BDBV GP and protected NHPs challenged with BDBV one week after vaccination [120]. This is BDBV-inclusive NHP evidence, but it remains preclinical and should not be interpreted as human BVD efficacy.

Human parainfluenza virus type 3 (HPIV3)-vectored pan-ebolavirus vaccine studies add an intranasal delivery dimension [121,122]. A trivalent HPIV3-vectored ebolavirus vaccine study reported antibody-mediated protective mechanisms [121], and a single-dose intranasal combination pan-ebolavirus approach provided BDBV ferret protection data [122]. These studies support further development of BDBV-inclusive mucosal vaccine approaches, but ferret protection and mechanistic antibody data do not substitute for BDBV NHP or human efficacy evidence. Recent vaccine reviews argue that filovirus vaccine development should move beyond a one-virus/one-vaccine paradigm while preserving rigorous virus-specific evidence requirements [15,32].

Protein, virus-like particle (VLP), nanoparticle, mRNA, viral-vector, and epitope-focused approaches differ substantially in their BDBV evidence. Protein-adjuvant vaccine studies explicitly included BDBV GP and measured BDBV-reactive humoral and cellular responses, but did not provide BDBV challenge protection [123]. Stabilized mucin-like-domain-deleted glycoprotein (GPΔmuc) and self-assembling protein nanoparticle (SApNP) work included BDBV GPΔmuc-WL^2^P^4^ trimers and BDBV GP-presenting SApNPs, together with BDBV pseudovirus neutralization readouts, but did not establish authentic BDBV protection [124]. Bivalent EBOV/SUDV VLP vaccination produced cross-neutralizing activity against BDBV GP-pseudotyped particles despite lacking BDBV GP as a vaccine antigen, which makes the BDBV relevance indirect [125]. EBOV glycoprotein ferritin nanoparticle (GP-Fer) immunofocusing similarly supports cross-neutralizing design principles but remains EBOV-antigen based and does not provide BDBV challenge data [126]. The BDBV heptad repeat 2-membrane-proximal external region (HR2-MPER) nanoparticle immunogen provides a useful negative design lesson: BDBV and EBOV GP-binding responses were induced, but meaningful BDBV neutralization and protection were not established [117]. By contrast, the lipid nanoparticle (LNP)-formulated mRNA vaccine encoding EBOV, BDBV, and SUDV GPs plus EBOV NP ([GPs+NP]@LNP) includes BDBV GP and provides BDBV-relevant rodent challenge and authentic BDBV immunodeficient-mouse exposure data, although no BDBV NHP or human efficacy data are available [127].

The BDBV vaccine pipeline changed rapidly during the 2026 outbreak. WHO-convened experts identified the single-dose rVSV Bundibugyo vaccine candidate developed by the International AIDS Vaccine Initiative (IAVI) as the most promising vaccine candidate, but estimated that it would likely require 7–9 months before clinical-trial assessment [23]. A second candidate, chimpanzee adenovirus Oxford 1 (ChAdOx1) Bundibugyo, developed by the University of Oxford and the Serum Institute of India, was considered potentially available within 2–3 months for efficacy assessment through a clinical trial, although additional animal data were still required to support and confirm further prioritization [23]. The Coalition for Epidemic Preparedness Innovations (CEPI) subsequently announced fast-track support for three BDBV vaccine candidates developed by IAVI, Moderna, Inc. (Cambridge, MA, USA), and the University of Oxford, with the Oxford candidate to be manufactured by the Serum Institute of India [128,129]. Separate from this outbreak-specific fast-track portfolio, the earlier CEPI/Oxford/Leipzig/Moderna multivalent filovirus program remains relevant as long-term BDBV-inclusive pipeline evidence because it targets EBOV, SUDV, BDBV, MARV, and potentially additional filoviruses, with immunogens to be tested on chimpanzee adenovirus Oxford vector (ChAdOx) and mRNA platforms [130]. These announcements are important for outbreak preparedness and trial readiness, but they are programmatic and regulatory-development evidence rather than BDBV protective-efficacy evidence.

Post-exposure vaccination is particularly relevant during outbreaks because many exposures are recognized only after contact with an infected patient or body of a deceased patient. In a post-exposure vaccination experiment, recombinant vesicular stomatitis virus lacking native glycoprotein and expressing BDBV glycoprotein (rVSVΔG/BDBV-GP) treatment 20–23 min after BDBV challenge resulted in survival of five of six cynomolgus macaques [131]. This is direct BDBV evidence, but it should be described as feasibility under artificial experimental conditions: treatment was administered within minutes of challenge, in a small treated cohort, with a single untreated concurrent control and reliance on historical controls, after a defined intramuscular laboratory challenge [131]. Occupational or community exposures in field outbreaks are usually recognized substantially later than this experimental interval. The field applicability of this approach after delayed exposure recognition therefore remains unknown, and no published BDBV post-exposure vaccination study has addressed delayed recognition intervals comparable to many occupational or community exposure investigations.

Post-exposure prophylaxis (PEP) should be considered separately from pre-exposure vaccination and treatment. The rVSVΔG/BDBV-GP study therefore represents direct but limited vaccine PEP evidence under artificially early treatment conditions [131]. Antiviral PEP evidence is indirect for BDBV: obeldesivir has post-exposure NHP efficacy against SUDV, EBOV, and MARV and was prioritized by WHO for BVD PEP evaluation, but no direct BDBV efficacy data were identified [23,76,77,78]. Remdesivir should be interpreted as a BDBV treatment-trial candidate supported by authentic-virus cell-culture susceptibility, not as BDBV PEP evidence [23,73]. Antibody or nanobody PEP remains candidate-specific and should be interpreted through the antibody evidence summarized in Table 4 rather than as a class-level claim [93,96,103,109,113]. No BDBV-specific studies were identified in the sources reviewed here that evaluate combined vaccine, antiviral, and antibody-based PEP. Such approaches should therefore be discussed only as research questions, not as evidence-supported BVD interventions.

The WHO Filovirus Research and Development Roadmap supports several vaccine priorities directly relevant to BDBV preparedness: advancement of additional filovirus vaccine candidates, exploration of pan-filovirus and multivalent approaches, harmonized immunogenicity and functional assays, immunobridging where validated correlates of protection exist, outbreak-ready clinical trial platforms, adaptive designs for vulnerable and high-risk groups, and resilient manufacturing and supply ecosystems [72]. These recommendations reinforce the need to move BDBV-inclusive candidates from antigen inclusion and small-animal evidence toward standardized, comparable, and outbreak-deployable evaluation. Vaccine and post-exposure prophylaxis evidence relevant to BDBV is summarized in Table 5, with pre-exposure vaccines, vaccine-development programs, vaccine PEP, antiviral PEP, and immunotherapy PEP or early-treatment concepts separated to avoid conflating protective efficacy, antigen design, programmatic prioritization, and post-exposure use.

## 6. Evidence Synthesis, Trial Readiness, and Implementation Implications

The BDBV countermeasure literature is heterogeneous, and its interpretation depends strongly on the experimental system. The strongest direct vaccine evidence comes from rVSV-BDBV-GP prophylactic NHP data [118] and rVSVΔG/BDBV-GP post-exposure NHP data [131]. However, both studies used small cohorts [118,131], and the post-exposure study used a treatment interval much shorter than realistic field recognition intervals [131]. Antibody-, protein-, peptide-, and nucleic-acid-based therapeutics provide a broader but uneven evidence landscape, ranging from BDBV survivor-derived antibody characterization [90,91] and in vitro mechanisms [92] to chimeric mouse models [94], surrogate-virus small-animal protection [95,109,113], direct BDBV NHP antibody treatment [93], and direct BDBV ferret and NHP antibody-cocktail protection [96]. Antiviral evidence is weaker and currently rests mainly on BDBV cell-culture susceptibility for remdesivir [73] plus extrapolation from other filoviruses for NHP antiviral efficacy [74,76,77,78].

Diagnostic evidence is conceptually strong but operationally uneven. Non-commercial broad conventional or dye-based filovirus RT-PCR/RT-qPCR assays [35,37,39,40], the RealStar^®^ assay suite spanning broad filovirus screening [38,41], ebolavirus screening without species differentiation [45], species-identification testing [50], BDBV-specific and differential species-specific RT-qPCR assays [4,48], EBOV-focused cartridge-based testing [24,25], and newer commercial closed systems with BDBV-inclusive *Orthoebolavirus* coverage [53,54,55,56,57,58,59] all have distinct roles. Targeted sequencing [4,35,37], agnostic metagenomic sequencing [4,14], and field-deployable genome sequencing [65] then provide species assignment, genomic confirmation, diagnostic-target assessment, and outbreak characterization. The practical lesson from the current outbreak is that a negative EBOV-directed assay result should trigger broader testing and sequencing when clinical and epidemiological suspicion remains high [12,19], rather than ending investigation. The problem is not simply analytical sensitivity; it is loss of negative predictive value when the assay target does not match the causative virus [4,24,25].

Clinical and immunological evidence reinforce the same need for species-specific interpretation. The published 2007–2008 and 2012 outbreak analyses show frequent non-specific systemic, gastrointestinal, and pain-related symptoms, variable fever documentation, and hemorrhage in only a subset of patients [6,10]. Human cytokine and serological data, together with rhesus macaque immune-signature data, suggest BDBV-specific host-response patterns that should not be collapsed into a generic EBOV-derived pathogenesis model [29,30]. This matters because therapeutic timing, antibody response, inflammatory markers, and survival correlates may differ by ebolavirus species and model.

Trial readiness is therefore a central preparedness gap [72]. The WHO Filovirus Research and Development Roadmap emphasizes outbreak-ready clinical trial platforms, pre-approved protocols, trained teams, interoperable data systems, adaptive trial designs, harmonized immunogenicity endpoints, harmonized serological and functional assays, reagent repositories, rapid data-sharing agreements, regulatory and ethical pathways, sample-access mechanisms, and inclusion of vulnerable populations [72]. These recommendations are highly relevant to BDBV because outbreaks are rare, geographically constrained, and often recognized under difficult field conditions. Without pre-positioned protocols, harmonized assays, sample-access agreements, and regional trial networks, promising BDBV countermeasure candidates may remain unevaluable during the short window in which cases occur.

During the 2026 BVD outbreak, WHO-convened experts recommended MBP134, maftivimab, and remdesivir for evaluation in clinical trials among confirmed BVD cases and also recommended evaluation of monoclonal antibody plus remdesivir combination therapy [23]. This prioritization should be interpreted as outbreak trial-readiness guidance rather than efficacy evidence [23]. It does not change the BDBV evidence hierarchy: MBP134/MBP134AF and BDBV289-N have stronger direct BDBV animal-efficacy evidence [93,96] than maftivimab [99,100], whereas remdesivir has BDBV cell-culture susceptibility evidence [73] and non-BDBV NHP efficacy evidence [74], but no BDBV animal or human efficacy data were identified.

Publicly available sources indicate that maftivimab was prioritized for clinical-trial evaluation [23] and that INMAZEB supply was available in the DRC [132], but no public source identified here confirmed maftivimab administration to BVD patients or reported preliminary BDBV efficacy outcomes [23,132,133]. The publicly described Charité case involved combined antiviral therapy, experimental therapies, supportive care, and clinical recovery after BDBV infection, but the administered agents were not named in the Charité statement [133]. Therefore, maftivimab should be interpreted as an operationally prioritized candidate [23] because of available supply [132] and regulatory coverage through its inclusion as a component of the approved INMAZEB^®^ combination product [98,132], not as a treatment with emerging BDBV clinical efficacy evidence [23,132,133].

Implementation constraints are equally important. Regulatory coordination also became part of the 2026 BVD outbreak response. EMA, African Medicines Agency (AMA), and African national regulatory authorities initiated joint engagement on clinical-trial designs and candidate medical countermeasures [134], while noting that EBOV-targeted countermeasures are unlikely to be effective for BDBV [134] and that customized vaccines, pan-filovirus monoclonal antibodies, antivirals, and well-designed randomized clinical trials are required [134]. Official DRC, Africa CDC, WHO, and CDC sources describe response needs that include strengthened surveillance, laboratory confirmation, contact tracing, infection prevention and control, safe care, safe burials, and risk communication [12,17,19,135], together with cross-border coordination, local border screening, logistics, regional coordination, and response deployment under conditions of insecurity and population mobility [17,21,135]. Very early post-exposure protection in an animal model [131] cannot be assumed to translate to field exposures identified late, during cross-border movement [21], or in areas where contact tracing and sample transport are difficult [12,17,135].

Deployment constraints also differ by modality. rVSV-based vaccines provide important rapid-protection data [118,120,131], but cold-chain requirements [136], outbreak logistics [12,17,135], access in insecure areas [17,135], and vaccine availability [23,128,129] remain major barriers. The licensed EBOV recombinant vesicular stomatitis virus lacking native glycoprotein and expressing Zaire ebolavirus glycoprotein (rVSVΔG-ZEBOV-GP) vaccine ERVEBO has stringent −80 °C to −60 °C storage requirements [136], illustrating the type of logistical constraint that may affect rVSV-based platforms if comparable formulations are used. Product-specific stability data would nevertheless be required before assuming identical cold-chain requirements for any BDBV vaccine candidate. Oral antivirals such as obeldesivir may have operational advantages for post-exposure or early treatment use [23,76,77,78], but BDBV efficacy has not been demonstrated [23]. Antibody, bispecific antibody, and nanobody products may offer rapid protection but require candidate-specific BDBV validation [93,96,109,113], manufacturability, dosing feasibility, and assessment of viral escape risk [99].

Equity and access should be treated as part of scientific preparedness, not as an afterthought. The WHO roadmap emphasizes equitable access to knowledge, tools, medical countermeasures, reagents, data, funding, clinical trials, diagnostics, therapeutics, and vaccines, as well as the need for community engagement and social-science integration [72]. Africa CDC’s regional coordination role is also directly relevant because BVD outbreak control and countermeasure evaluation require African public-health leadership, cross-border coordination, and sustained capacity in the countries and regions most likely to face future BDBV outbreaks [21].

Actionable priorities follow directly from this evidence hierarchy: broad diagnostic algorithms that do not stop at EBOV-negative results; sequencing capacity for species confirmation and assay-target assessment; authentic BDBV validation of antiviral and antibody candidates; realistic post-exposure timing studies; standardized comparison of antiviral, antibody, nanobody, and vaccine candidates; harmonized immunological assays; and pre-approved, regionally led clinical and data-sharing frameworks [72]. Human outbreak observations, authentic BDBV NHP and ferret studies, surrogate animal models, authentic-virus assays, minigenome systems, GP-pseudotyped lentiviral particle neutralization assays, binding studies, antigen-inclusion data, and extrapolation from other filoviruses should remain explicitly separated because they answer different preparedness questions.

## 7. Conclusions

Preparedness for BVD remains limited despite repeated outbreaks and substantial preclinical progress. Diagnostic development has shown that broad filovirus detection and sequencing can complement species-specific assays, but implementation remains uneven. The May 2026 outbreak demonstrates the practical consequences of assay-target mismatch and the value of broad RT-PCR, diagnostic escalation, sequencing-based confirmation, and regional genomic and public-health coordination. Antiviral evidence is sparse and largely mechanistic or cell-culture based. Antibody-, protein-, peptide-, and nucleic-acid-based therapeutics include survivor-derived antibodies, pan-ebolavirus antibody cocktails, bispecific antibodies, mRNA-encoded antibodies, nanobodies, and computationally optimized antibody variants, but the strength of BDBV-specific evidence varies widely. Vaccines, especially rVSV-BDBV-GP approaches, currently provide the most substantial direct BDBV protection data, including limited post-exposure evidence in NHPs. Newer mRNA vaccine platforms, bispecific antibody studies, nanobody approaches, and epitope-focused immunogen designs expand the pipeline, but most remain early, surrogate-model based, or preclinical and require validation in higher-order models.

The main research priorities are authentic BDBV validation of antiviral and antibody candidates, realistic post-exposure timing studies, standardized comparison of antiviral, antibody, nanobody, and vaccine candidates, expanded evaluation of BDBV-inclusive multivalent vaccines, harmonized diagnostic and immunological assays, ethical sample-access and data-sharing pathways, and clinical trial platforms that can be activated rapidly during outbreaks. BVD preparedness requires neither exclusive BDBV-specific development nor uncritical reliance on broad platforms. It requires evidence-based integration of diagnostics capable of identifying BDBV early, regionally coordinated outbreak response, and countermeasures whose breadth has been demonstrated rather than assumed.

## Figures and Tables

**Table 2:** Sequencing-based approaches relevant to Bundibugyo virus species identification, genomic confirmation, and outbreak characterization. Abbreviations: BDBV, Bundibugyo virus; L, large polymerase gene; NGS, next-generation sequencing; NP, nucleoprotein gene; RNA, ribonucleic acid; RT-PCR, conventional endpoint reverse transcription polymerase chain reaction.

Approach	Format	Target or Input	Diagnostic Role	BDBV Relevance	Main Limitation	Source
Sanger sequencing of broad RT-PCR amplicons	Amplicon sequencing	L or NP product, assay-dependent	Species identification after broad RT-PCR	Supports BDBV identification after broad RT-PCR positivity.	Requires a positive amplicon and sequencing capacity.	[4,35,37]
Whole or near-whole genome sequencing	NGS	Whole or near-whole genome, viral RNA	Genomic confirmation, lineage assignment, outbreak reconstruction, and diagnostic-target monitoring	Relevant to BDBV genomic confirmation and reconstruction of the 2012 outbreak.	Requires sequencing capacity, contamination control, bioinformatics, and interpretation infrastructure.	[4,9,11,14]
Agnostic or semi-agnostic metagenomic sequencing	NGS-based diagnostic escalation	No fixed target	Investigation of unknown or divergent agents	Relevant when targeted assays are negative or inconclusive. Contributed to original BDBV recognition.	Lower sensitivity than optimized targeted assays in some settings. Requires robust controls and analysis.	[4,14]

**Table 3:** Small-molecule antiviral candidates and antiviral-susceptibility evidence relevant to Bundibugyo virus. Abbreviations: BDBV, Bundibugyo virus; BVD, Bundibugyo virus disease; EBOV, Ebola virus; HepG2, human hepatocellular carcinoma cell line; MARV, Marburg virus; MEURI, Monitored Emergency Use of Unregistered and Investigational Interventions; NHP, nonhuman primate; nM, nanomolar; RNA, ribonucleic acid; SUDV, Sudan virus; T562A, threonine-to-alanine substitution at polymerase residue 562.

Type/Class	Candidate	Evidence Category	BDBV-Relevant Evidence	Main Caveat	Interpretation	Key Source
Nucleotide analog prodrug/viral RNA polymerase inhibitor	Remdesivir/GS-5734	Authentic BDBV in vitro	BDBV minigenome system and authentic-virus HepG2 inhibition; 90% effective concentration 109.6 nM for BDBV	No authentic BDBV animal post-exposure prophylaxis or human BVD efficacy data	BDBV cell-culture susceptibility signal only	[73]
Polymerase susceptibility/resistance context	Remdesivir resistance context	Sequence/polymerase susceptibility evidence	T562A occurs naturally in BDBV and Taï Forest virus	Functional effect in BDBV polymerase has not been directly established	Supports isolate-level validation	[75]
Orally bioavailable nucleoside analog prodrug	Obeldesivir/GS-5245	Extrapolation from other filoviruses	No direct BDBV efficacy data identified in the sources reviewed here	NHP evidence is from SUDV, MARV, and EBOV models	Broad filovirus antiviral candidate; BDBV efficacy unproven	[76,77,78]
Nucleoside analog/viral RNA polymerase inhibitor	Galidesivir/BCX4430	Extrapolation from other filoviruses; 2026 MEURI deployment	No direct BDBV efficacy data identified; Uganda reportedly approved compassionate use under a MEURI protocol during the 2026 BVD outbreak, with prospective clinical, safety, and virological data collection	EBOV and MARV preclinical/NHP evidence does not establish BDBV efficacy; MEURI use is not regulatory approval or efficacy evidence	Broad-spectrum filovirus antiviral candidate; potential source of rare human BVD outbreak data	[79,80,81,82]
Nucleoside analog antiviral	Favipiravir	Extrapolation from other filoviruses	No strong direct BDBV evidence identified	EBOV and other filovirus data do not establish BDBV efficacy	Include cautiously	[83]

**Table 4:** Antibody-, nanobody-, peptide-, and nucleic-acid-based therapeutic candidates relevant to Bundibugyo virus. Abbreviations: ADI, antibody discovery/optimization identifier; BDBV, Bundibugyo virus; BST2, bone marrow stromal antigen 2; BVD, Bundibugyo virus disease; EBOV, Ebola virus; GP, glycoprotein; IFNAR−/−, type I interferon receptor-deficient; LC, light chain; mAb, monoclonal antibody; mAbs, monoclonal antibodies; mRNA, messenger ribonucleic acid; NHP, nonhuman primate; pNL4.3-Luc-E−R−, luciferase-expressing, envelope-defective lentiviral reporter construct; rEBOV-515 and rEBOV-442, broadly neutralizing human monoclonal antibodies used in a pan-ebolavirus antibody cocktail; rVSV, recombinant vesicular stomatitis virus; STAT1, signal transducer and activator of transcription 1; SUDV, Sudan virus; VSV, vesicular stomatitis virus.

Type/Class	Candidate	Evidence Category	Bundibugyo Virus-Relevant Evidence	Main Caveat	Interpretation	Key Source
BDBV survivor-derived mAbs	BDBV survivor-derived monoclonal antibodies	Human BVD-derived antibody evidence	Antibodies isolated from BDBV survivors	Breadth varies by antibody	Demonstrates inducible cross-reactive humoral responses	[90]
BDBV survivor-derived mAb	BDBV289-N	Authentic BDBV NHP challenge	6/6 treated macaques survived after treatment as late as 8 days after challenge	6/10 untreated controls also survived; no human BVD data	Important direct BDBV antibody dataset, but survival efficacy should not be overstated	[93]
BDBV survivor-derived cross-reactive mAb	BDBV223	BDBV survivor antibody plus in vitro mechanism and surrogate animal model	Neutralizes BDBV and EBOV; protected 3/5 STAT1 knockout mice challenged with EBOV/BDBV-GP when administered 24 h after challenge; blocks BDBV intercellular spread in a BST2/tetherin-dependent mechanism	Does not neutralize SUDV; chimeric mouse and in vitro spread models are not authentic BDBV NHP disease	Cross-reactive across two species; useful BDBV antibody/model/mechanistic evidence	[91,92,94]
Pan-ebolavirus mAb cocktail	MBP134AF	Authentic BDBV ferret and NHP challenge	Direct BDBV ferret and cynomolgus macaque protection data; 5/6 treated macaques survived versus 0/3 phosphate-buffered saline controls	No human BVD data; preclinical model timing and dose constraints remain	One of the strongest BDBV-relevant antibody-cocktail candidates, but still preclinical	[96]
Broad ebolavirus mAb pair/cocktail	1C3/1C11	BDBV-GP surrogate animal model plus EBOV/SUDV NHP evidence	1C11 alone gave 80% protection and 1C3/1C11 gave 100% protection in STAT1 knockout mice challenged with EBOV/BDBV-GP	No authentic BDBV animal challenge; NHP protection was EBOV/SUDV, not BDBV	Relevant broad-antibody and BDBV-GP surrogate evidence	[103]
Broad ebolavirus mAb cocktail platform	rEBOV-515/rEBOV-442	Broad ebolavirus antibody-cocktail platform	Broad ebolavirus protective therapy platform with relevance to medically important ebolaviruses	BDBV-specific interpretation depends on exact assay and model endpoints	Important conserved-site antibody-cocktail platform	[102]
Conserved-epitope mAbs	Macaque-derived conserved-epitope monoclonal antibodies	Broad filovirus antibody-discovery platform	Cross-reactive macaque monoclonal antibodies targeting conserved filovirus GP epitopes; some broad neutralization and mouse protection	No direct BDBV challenge protection	Useful early pan-filovirus antibody-discovery evidence; lower priority than later BDBV-specific datasets	[104]
Broad mAb combination	FVM04/CA45	Extrapolation from other filoviruses	EBOV and SUDV protection in NHPs	Direct BDBV protection should not be assumed	Useful comparison for broad antibody design	[105]
Bispecific mAb	EBOV/SUDV bispecific antibody	Extrapolation from other filoviruses	No direct BDBV efficacy claim	EBOV/SUDV-focused protection	Supports dual-epitope engineering concept	[106]
Pan-ebolavirus mAb cocktail	Pan-ebolavirus monoclonal antibody cocktail	Broad ebolavirus antibody platform	BDBV relevance depends on in vitro breadth	Protection reported for EBOV/SUDV models	Breadth-engineering, not direct BDBV protection	[107]
Pan-ebolavirus/pan-filovirus bispecific mAbs	Pan-ebolavirus and pan-filovirus bispecific antibodies	Broad bispecific antibody platform	BDBV relevance construct-dependent	Broad activity does not equal BDBV protection	Platform relevance, construct-specific interpretation	[108]
Engineered bispecific mAbs	S1+5 and related rEBOV-515/1C3-derived bispecific antibodies	BDBV surrogate-virus animal model plus authentic-virus neutralization	Improved neutralization against rVSV expressing BDBV GP; complete or high protection in rVSV-BDBV IFNAR−/− mouse challenge depending on construct; authentic BDBV neutralization assessed	No authentic BDBV animal challenge; authors note need for authentic BDBV studies	Relevant surrogate small-animal bispecific antibody evidence; include but do not overstate	[109]
Computationally optimized mAb variants	ADI-15878/ADI-15946 optimized variants	BDBV-GP-pseudotyped lentiviral reporter-particle and computational antibody-engineering evidence	W32G-LC improved neutralization of pNL4.3-Luc-E−R− lentiviral reporter particles pseudotyped with EBOV, BDBV, or SUDV GP; ADI-15946 variants retained activity against particles bearing EBOV or BDBV GP while improving neutralization of particles bearing SUDV GP	GP-pseudotyped lentiviral reporter-particle and binding-stage evidence; no authentic BDBV neutralization, animal protection, or human data	Relevant antibody-engineering evidence only	[111]
mRNA-encoded mAb	2G1 mRNA antibody	mRNA-encoded antibody platform	Pan-orthoebolavirus relevance reported	No human BVD data	Emerging modality requiring assay-specific BDBV clarification	[110]
Synthetic-library nanobody	BDBV-Nb02 nanobody	BDBV-GP-pseudotyped lentiviral reporter-particle evidence	BDBV and EBOV GP-pseudotyped lentiviral particle neutralization	No SUDV activity; no authentic-virus or animal protection data	Rapid discovery candidate requiring validation	[112]
Nanobody/bispecific nanobody	1A10/BA2/BA2-1A10	Nanobody/bispecific nanobody, surrogate animal model	EBOV, SUDV, and BDBV in vitro neutralization; rodent protection including VSV-BDBV model	No authentic BDBV NHP or human data	Strongest nanobody-based BDBV-relevant protection data identified here	[113]
Epitope-focused peptide/immunogen-design approach	Heptad repeat 2-membrane-proximal external region/epitope-focused approaches	Epitope/immunogen design	BDBV heptad repeat 2-membrane-proximal external region design can elicit BDBV and EBOV GP-binding antibodies	Neutralization and protection remain weak or absent in current immunogen studies	Relevant mainly to future antibody and vaccine design	[117]

**Table 5:** Vaccines and post-exposure prophylaxis candidates relevant to Bundibugyo virus. Entries are grouped by intended use and ranked within each group by directness of BDBV evidence. Abbreviations: [GPs+NP]@LNP, lipid nanoparticle-formulated mRNA vaccine encoding Ebola virus, Bundibugyo virus, and Sudan virus glycoproteins plus Ebola virus nucleoprotein; BDBV, Bundibugyo virus; BVD, Bundibugyo virus disease; CEPI, Coalition for Epidemic Preparedness Innovations; ChAdOx, chimpanzee adenovirus Oxford vector; ChAdOx1, chimpanzee adenovirus Oxford 1; CIEBOV, Côte d’Ivoire ebolavirus; EBOV, Ebola virus; GP, glycoprotein; GP-Fer, glycoprotein ferritin nanoparticle; GPΔmuc, mucin-like-domain-deleted glycoprotein; GS-5245, obeldesivir; HPIV3, human parainfluenza virus type 3; HR2-MPER, heptad repeat 2-membrane-proximal external region; IAVI, International AIDS Vaccine Initiative, New York, NY, USA; IFNAR−/−, type I interferon receptor-deficient; LNP, lipid nanoparticle; LU-IDD, Leipzig University Institute for Drug Discovery, Leipzig, Germany; MOD, Moderna, Inc., Cambridge, MA, USA; mRNA, messenger ribonucleic acid; NHP, nonhuman primate; NP, nucleoprotein; OU, University of Oxford, Oxford, UK; PEP, post-exposure prophylaxis; PrEV, pre-exposure vaccination/prophylactic vaccine; rVSV, recombinant vesicular stomatitis virus; rVSV-BDBV-GP, recombinant vesicular stomatitis virus expressing Bundibugyo virus glycoprotein; rVSV-CIEBOV-GP, recombinant vesicular stomatitis virus expressing Côte d’Ivoire ebolavirus glycoprotein; rVSV-EBOV-GP, recombinant vesicular stomatitis virus expressing Ebola virus glycoprotein; rVSV-Filo, recombinant vesicular stomatitis virus-based filovirus vaccine formulation; rVSV-SUDV-GP, recombinant vesicular stomatitis virus expressing Sudan virus glycoprotein; rVSVΔG/BDBV-GP, recombinant vesicular stomatitis virus lacking native glycoprotein and expressing Bundibugyo virus glycoprotein; SApNP, self-assembling protein nanoparticle; SII, Serum Institute of India Pvt. Ltd., Pune, India; SUDV, Sudan virus; VLP, virus-like particle; VSVΔG-BDBV-GP, vesicular stomatitis virus lacking native glycoprotein and pseudotyped with Bundibugyo virus glycoprotein.

Intervention Type	Candidate or Platform	Evidence Category	Bundibugyo Virus-Relevant Evidence	Main Caveat	Interpretation	Key Source
**Vaccines and vaccine-development platforms**						
PrEV	rVSV vaccine expressing BDBV GP	Authentic BDBV NHP challenge	rVSV-BDBV-GP protected all vaccinated macaques against BDBV challenge without overt disease or detectable viremia in the protected cohort	Small cohort; no human BVD data; not licensed for BVD	Strongest species-specific BDBV prophylactic vaccine evidence, but still preclinical	[118]
PrEV	Quadrivalent VesiculoVax rVSV-Filo vaccine	Authentic BDBV NHP challenge	Quadrivalent rVSV-Filo vaccine included BDBV GP and protected macaques challenged with BDBV 7 days after vaccination	Small NHP cohorts; rapid 7-day prechallenge schedule is not equivalent to field efficacy	Strong BDBV-inclusive multivalent NHP evidence; preclinical outbreak-readiness signal, not licensure-level evidence	[120]
PrEV	Heterologous rVSV-SUDV-GP/rVSV-EBOV-GP prime-boost	Authentic BDBV NHP challenge	Short heterologous rVSV prime-boost protected macaques against BDBV challenge, whereas the single blended heterologous strategy failed	Requires sequential prime-boost; protection is schedule-dependent and does not use BDBV GP	Shows that cross-species rVSV protection can occur, but is less direct than BDBV-GP-containing vaccination	[118]
PrEV	Monovalent heterologous rVSV-EBOV-GP vaccine	Authentic BDBV NHP challenge	Single rVSV-EBOV-GP vaccination gave partial heterologous protection against BDBV challenge; rVSV-CIEBOV-GP did not clearly outperform controls	Heterologous protection was incomplete and antigen-dependent	Useful warning that rVSV platform similarity does not guarantee BDBV protection	[119]
PrEV	HPIV3-vectored trivalent pan-ebolavirus vaccine	BDBV ferret challenge evidence	Single intranasal trivalent HPIV3 vaccine expressing EBOV, SUDV, and BDBV GPs protected ferrets against lethal BDBV challenge	Ferret model only; no BDBV NHP or human efficacy data	Strong BDBV-relevant mucosal vaccine evidence below NHP-level evidence	[122]
PrEV	Multivalent [GPs+NP]@LNP mRNA vaccine	BDBV surrogate-virus animal model plus authentic BDBV immunodeficient-mouse exposure	Encodes EBOV, BDBV, and SUDV GPs plus EBOV NP; BDBV-relevant testing included VSVΔG-BDBV-GP challenge and reduced viral loads after authentic BDBV exposure in IFNAR−/− mice	Rodent and immunodeficient-mouse data only; no BDBV NHP or human efficacy data	Strong BDBV-inclusive mRNA vaccine-platform evidence, but still early preclinical	[127]
PrEV	Protein-adjuvant EBOV/SUDV/BDBV GP vaccines	BDBV antigen-inclusion and immunogenicity evidence	Recombinant BDBV GP was included in mono- and multivalent adjuvanted GP formulations; mouse studies measured BDBV GP-binding, neutralization, and T-cell recall responses	Mouse immunogenicity only; no BDBV challenge protection	BDBV-inclusive immunogenicity evidence; cannot substitute for protection data	[123]
PrEV	Stabilized BDBV GPΔmuc trimer/BDBV GP-presenting SApNP vaccines	BDBV antigen-design, antigenicity, and pseudovirus-neutralization evidence	BDBV GPΔmuc-WL^2^P^4^ trimers and BDBV GP-presenting SApNPs were designed and characterized; BDBV pseudovirus was included in cross-neutralization readouts	No authentic BDBV challenge protection; in vivo immunogenicity was not a BDBV-protection study	Explicit BDBV rational antigen-design evidence, not BDBV efficacy evidence	[124]
PrEV	BDBV HR2-MPER epitope-focused nanoparticle	BDBV epitope-focused immunogen evidence	BDBV HR2-MPER epitope was transplanted onto scaffold proteins and displayed on nanoparticles; rabbit immunization induced BDBV and EBOV GP-binding antibodies	No meaningful BDBV neutralization except limited activity in one peptide-immunized animal; no protection data	Important negative/limiting design evidence: binding breadth did not translate into robust neutralization	[117]
PrEV	Bivalent EBOV/SUDV VLP vaccine	BDBV pseudovirus-neutralization and NHP immunogenicity evidence	EBOV/SUDV GP VLP vaccination generated sera with cross-neutralization against BDBV GP-pseudotyped particles; rhesus macaques developed humoral and cellular responses to the bivalent VLP vaccine	BDBV GP was not part of the vaccine; no BDBV challenge protection	Cross-neutralization signal only; weaker than BDBV-antigen-containing vaccine evidence	[125]
PrEV	EBOV GP-Fer immunofocusing vaccine candidates	BDBV/SUDV cross-neutralization from EBOV-based immunogen design	Hyperglycosylated EBOV GP-Fer immunogens elicited cross-neutralizing activity against BDBV and SUDV in mice more consistently than wild-type EBOV GP-Fer	EBOV-based design; no BDBV antigen, authentic BDBV challenge, or BDBV protection data	Relevant universal-vaccine design concept, but indirect for BDBV	[126]
Vaccine-development program	CEPI 2026 BDBV fast-track vaccine portfolio	Official program/outbreak trial-readiness evidence	Portfolio includes IAVI rVSV-BDBV, MOD mRNA-BDBV, and OU/SII ChAdOx1-BDBV candidates selected for accelerated development during the 2026 outbreak	No BDBV clinical efficacy data; candidates remain investigational and at different readiness stages	Important outbreak-response context; product prioritization is not protective-efficacy evidence	[23,128,129]
Vaccine-development program	CEPI/OU/LU-IDD/MOD multivalent ChAdOx/mRNA filovirus program	Official program/design-stage pipeline evidence	Long-term multivalent filovirus program targets EBOV, SUDV, BDBV, MARV, and potentially additional filoviruses; LU-IDD immunogens are to be tested on OU ChAdOx and MOD mRNA platforms	Program-level evidence only; no product-level BDBV immunogenicity or protection data	Long-term BDBV-inclusive pipeline context, not countermeasure efficacy evidence	[130]
**Post-exposure prophylaxis and early post-exposure interventions**						
Vaccine PEP	rVSVΔG/BDBV-GP vaccine	Authentic BDBV NHP post-exposure challenge	5/6 macaques survived when rVSVΔG/BDBV-GP was administered 20–23 min after BDBV challenge	Treatment interval was minutes after challenge; small treated cohort, single concurrent control, and historical controls; no human data	Direct BDBV vaccine PEP proof of concept under artificial timing; field applicability after delayed exposure recognition remains unproven	[131]
Antiviral PEP	Obeldesivir/GS-5245	Extrapolation from other filoviruses plus WHO BVD PEP prioritization	Oral obeldesivir protected NHPs after SUDV, EBOV, and MARV exposure and was prioritized by WHO for BVD PEP evaluation among contacts	No direct BDBV efficacy data; PEP utility depends on rapid contact identification and dosing after exposure	Operationally attractive oral PEP candidate, but BDBV efficacy remains unproven	[23,76,77,78]
Immunotherapy PEP/early treatment	BDBV289-N, MBP134AF, 1C3/1C11, engineered bispecific antibodies, and nanobody/bispecific candidates	Candidate-specific antibody or nanobody evidence	BDBV289-N and MBP134AF have direct BDBV treatment/protection datasets; 1C3/1C11, engineered bispecific antibodies, and BA2-1A10 provide BDBV-relevant surrogate or small-animal protection evidence	Not a unified PEP regimen; timing, dose, route, escape risk, and authentic-virus protection remain candidate-specific	Should be interpreted through Table 4 candidate-specific evidence, not as class-level antibody PEP efficacy	[93,96,103,109,113]

## Data Availability

No new data were created or analyzed in this study. Publicly available reports, genome resources, and publications discussed in this review are cited in the reference list.

## Abbreviations

The following abbreviations are used in this manuscript:

Africa CDC	Africa Centres for Disease Control and Prevention
AMA	African Medicines Agency
BCX4430	galidesivir
BDBV	Bundibugyo virus (*Orthoebolavirus bundibugyoense*)
bp	base pairs
BSL-2	biosafety level 2
BSL-4	biosafety level 4
BST2	bone marrow stromal antigen 2
BVD	Bundibugyo virus disease
CCHFV	Crimean–Congo hemorrhagic fever virus
CDC	Centers for Disease Control and Prevention
CEPI	Coalition for Epidemic Preparedness Innovations
ChAdOx	chimpanzee adenovirus Oxford vector
ChAdOx1	chimpanzee adenovirus Oxford 1
CIEBOV	Côte d’Ivoire ebolavirus
COVID-19	coronavirus disease 2019
DRC	Democratic Republic of the Congo
EBOV	Ebola virus (*Orthoebolavirus zairense*)
EMA	European Medicines Agency
EUAL	Emergency Use Assessment and Listing
EUL	Emergency Use Listing
FDA	United States Food and Drug Administration
GP	glycoprotein
GP2	glycoprotein subunit 2
GP-Fer	glycoprotein ferritin nanoparticle
GPΔmuc	mucin-like-domain-deleted glycoprotein
[GPs+NP]@LNP	lipid nanoparticle-formulated mRNA vaccine encoding Ebola virus, Bundibugyo virus, and Sudan virus glycoproteins plus Ebola virus nucleoprotein
GS-5245	obeldesivir
GS-5734	remdesivir
HepG2	human hepatocellular carcinoma cell line
HPIV3	human parainfluenza virus type 3
HR2-MPER	heptad repeat 2-membrane-proximal external region
IAVI	International AIDS Vaccine Initiative
ICTV	International Committee on Taxonomy of Viruses
IFNAR−/−	type I interferon receptor-deficient
IFU	instructions for use
INMAZEB	atoltivimab/maftivimab/odesivimab-ebgn
INRB	Institut National de Recherche Biomédicale
IPPS	International Pandemic Preparedness Secretariat
L	large polymerase gene
LC	light chain
LNP	lipid nanoparticle
LoD95	95% limit of detection
LU-IDD	Leipzig University Institute for Drug Discovery
mAb	monoclonal antibody
mAb114	monoclonal antibody 114
mAbs	monoclonal antibodies
MARV	Marburg virus
MEURI	Monitored Emergency Use of Unregistered and Investigational Interventions
MGB	minor groove binder
MIQE	Minimum Information for Publication of Quantitative Real-Time PCR Experiments
MOD	Moderna, Inc.
MPER	membrane-proximal external region
mRNA	messenger ribonucleic acid
NCBI	National Center for Biotechnology Information
NGS	next-generation sequencing
NHP	nonhuman primate
nM	nanomolar
NP	nucleoprotein
ONT	Oxford Nanopore Technologies plc, Oxford, UK
OU	University of Oxford
PALM	Pamoja Tulinde Maisha
PCR	polymerase chain reaction
PEP	post-exposure prophylaxis
pNL4.3-Luc-E−R−	luciferase-expressing, envelope-defective lentiviral reporter construct
PoC	point of care
PrEV	pre-exposure vaccination/prophylactic vaccine
RDT	rapid diagnostic test
REGN3479	maftivimab
REGN-EB3	atoltivimab/maftivimab/odesivimab-ebgn
RESTV	Reston virus (*Orthoebolavirus restonense*)
RNA	ribonucleic acid
RT-LAMP	reverse transcription loop-mediated isothermal amplification
RT-PCR	reverse transcription polymerase chain reaction
RT-qPCR	real-time reverse transcription quantitative polymerase chain reaction
rVSV	recombinant vesicular stomatitis virus
rVSV-BDBV-GP	recombinant vesicular stomatitis virus expressing Bundibugyo virus glycoprotein
rVSV-EBOV-GP	recombinant vesicular stomatitis virus expressing Ebola virus glycoprotein
rVSV-Filo	recombinant vesicular stomatitis virus-based filovirus vaccine formulation
rVSVΔG/BDBV-GP	recombinant vesicular stomatitis virus lacking native glycoprotein and expressing Bundibugyo virus glycoprotein
rVSVΔG-ZEBOV-GP	recombinant vesicular stomatitis virus lacking native glycoprotein and expressing Zaire ebolavirus glycoprotein
rVSV-SUDV-GP	recombinant vesicular stomatitis virus expressing Sudan virus glycoprotein
RUO	research use only
SAM	sterile alpha motif
SApNP	self-assembling protein nanoparticle
SII	Serum Institute of India Pvt. Ltd.
STAT1	signal transducer and activator of transcription 1
SUDV	Sudan virus (*Orthoebolavirus sudanense*)
TAFV	Taï Forest virus (*Orthoebolavirus taiense*)
TIM-1	T-cell immunoglobulin and mucin domain 1
T562A	threonine-to-alanine substitution at polymerase residue 562
VHF	viral hemorrhagic fever
VLP	virus-like particle
VSV	vesicular stomatitis virus
VSVΔG-BDBV-GP	vesicular stomatitis virus lacking native glycoprotein and pseudotyped with Bundibugyo virus glycoprotein
WHO	World Health Organization

## References

[1] Biedenkopf N., Bukreyev A., Chandran K., Di Paola N., Formenty P.B.H., Griffiths A., Hume A.J., Mühlberger E., Netesov S.V., Palacios G. (2024). ICTV Virus Taxonomy Profile: Filoviridae 2024. J. Gen. Virol..

[2] Kuhn J.H., Adachi T., Adhikari N.K.J., Arribas J.R., Bah I.E., Bausch D.G., Bhadelia N., Borchert M., Brantsæter A.B., Brett-Major D.M. (2019). New filovirus disease classification and nomenclature. Nat. Rev. Microbiol..

[3] Jacob S.T., Crozier I., Fischer W.A., Hewlett A., Kraft C.S., Vega M.A., Soka M.J., Wahl V., Griffiths A., Bollinger L. (2020). Ebola virus disease. Nat. Rev. Dis. Primers.

[4] Towner J.S., Sealy T.K., Khristova M.L., Albariño C.G., Conlan S., Reeder S.A., Quan P.L., Lipkin W.I., Downing R., Tappero J.W. (2008). Newly discovered ebola virus associated with hemorrhagic fever outbreak in Uganda. PLoS Pathog..

[5] Wamala J.F., Lukwago L., Malimbo M., Nguku P., Yoti Z., Musenero M., Amone J., Mbabazi W., Nanyunja M., Zaramba S. (2010). Ebola hemorrhagic fever associated with novel virus strain, Uganda, 2007–2008. Emerg. Infect. Dis..

[6] Roddy P., Howard N., Van Kerkhove M.D., Lutwama J., Wamala J., Yoti Z., Colebunders R., Palma P.P., Sterk E., Jeffs B. (2012). Clinical manifestations and case management of Ebola haemorrhagic fever caused by a newly identified virus strain, Bundibugyo, Uganda, 2007–2008. PLoS ONE.

[7] Centers for Disease Control and Prevention Outbreak History. https://www.cdc.gov/ebola/outbreaks/index.html.

[8] MacNeil A., Farnon E.C., Wamala J., Okware S., Cannon D.L., Reed Z., Towner J.S., Tappero J.W., Lutwama J., Downing R. (2010). Proportion of deaths and clinical features in Bundibugyo Ebola virus infection, Uganda. Emerg. Infect. Dis..

[9] Albariño C.G., Shoemaker T., Khristova M.L., Wamala J.F., Muyembe J.J., Balinandi S., Tumusiime A., Campbell S., Cannon D., Gibbons A. (2013). Genomic analysis of filoviruses associated with four viral hemorrhagic fever outbreaks in Uganda and the Democratic Republic of the Congo in 2012. Virology.

[10] Kratz T., Roddy P., Tshomba Oloma A., Jeffs B., Pou Ciruelo D., de la Rosa O., Borchert M. (2015). Ebola virus disease outbreak in Isiro, Democratic Republic of the Congo, 2012: Signs and symptoms, management and outcomes. PLoS ONE.

[11] Hulseberg C.E., Kumar R., Di Paola N., Larson P., Nagle E.R., Richardson J., Hanson J., Wauquier N., Fair J.N., Makuwa M. (2021). Molecular analysis of the 2012 Bundibugyo virus disease outbreak. Cell Rep. Med..

[12] World Health Organization Disease Outbreak News: Ebola Disease Caused by Bundibugyo Virus—Democratic Republic of the Congo and Uganda. https://www.who.int/emergencies/disease-outbreak-news/item/2026-DON602.

[13] Burk R., Bollinger L., Johnson J.C., Wada J., Radoshitzky S.R., Palacios G., Bavari S., Jahrling P.B., Kuhn J.H. (2016). Neglected filoviruses. FEMS Microbiol. Rev..

[14] Di Paola N., Sanchez-Lockhart M., Zeng X., Kuhn J.H., Palacios G. (2020). Viral genomics in Ebola virus research. Nat. Rev. Microbiol..

[15] Marzi A., Feldmann H. (2024). Filovirus vaccines as a response paradigm for emerging infectious diseases. npj Vaccines.

[16] Yamaoka S., Ebihara H. (2021). Pathogenicity and virulence of ebolaviruses with species- and variant-specificity. Virulence.

[17] Ministère de la Santé Publique, Hygiène et Prévoyance Sociale, République Démocratique du Congo Déclaration de la 17ème Épidémie de la Maladie à Virus Ebola dans les Zones de Santé de Rwampara, Mongbwalu et Bunia dans la Province d’Ituri. https://administration.sante.gouv.cd/wp-content/uploads/2026/05/Declaration-de-la-17e-Epidemie-de-la-maladie-a-virus-Ebola-dans-les-zones-de-sante-de-Rwampara-Mongwalu-et-Bunia-dans-la-province-dIturi.pdf.

[18] World Health Organization Epidemic of Ebola Disease Caused by Bundibugyo Virus in the Democratic Republic of the Congo and Uganda Determined a Public Health Emergency of International Concern. https://www.who.int/news/item/17-05-2026-epidemic-of-ebola-disease-in-the-democratic-republic-of-the-congo-and-uganda-determined-a-public-health-emergency-of-international-concern.

[19] Centers for Disease Control and Prevention Ebola Disease: Current Situation. https://www.cdc.gov/ebola/situation-summary/index.html.

[20] Rigby J., Le Poidevin O. (2026). Ebola Likely Circulating in Congo for Two Months, Outbreak to Grow, WHO Says. Reuters. https://www.reuters.com/business/healthcare-pharmaceuticals/who-says-139-suspected-ebola-deaths-congo-outbreak-numbers-expected-rise.

[21] Africa Centres for Disease Control and Prevention Africa CDC Calls Urgent Regional Coordination Meeting Following Ebola Outbreak in Ituri Province, DRC. https://africacdc.org/news-item/africa-cdc-calls-for-urgent-regional-coordination-meeting-following-ebola-virus-disease-outbreak-in-ituri-province-drc/.

[22] BEACON (2026). Bundibugyo Virus Disease Outbreak, DRC, Uganda, and South Sudan: Nearly 600 Suspected Cases Reported; Suspected Case Under Investigation in South Sudan; Investigational Vaccine and Therapeutic Discussions Ongoing. BEACON. https://beaconbio.org/en/report/?reportid=0dcdca95-b232-4565-a660-f9a608581e5c&eventid=1a9a0ef4-262f-45a1-9018-aa8f99d93a7c.

[23] World Health Organization Experts Convened by WHO Advise on Candidate Treatments and Vaccines for Ebola Disease Caused by Bundibugyo Virus. https://www.who.int/news/item/28-05-2026-experts-convened-by-who-advise-on-candidate-treatments-and-vaccines-for-ebola-disease-caused-by-bundibugyo-virus.

[24] Pinsky B.A., Sahoo M.K., Sandlund J., Kleman M., Kulkarni M., Grufman P., Nygren M., Kwiatkowski R., Baron E.J., Tenover F. (2015). Analytical performance characteristics of the Cepheid GeneXpert Ebola assay for the detection of Ebola virus. PLoS ONE.

[25] Semper A.E., Broadhurst M.J., Richards J., Foster G.M., Simpson A.J., Logue C.H., Kelly J.D., Miller A., Brooks T.J., Murray M. (2016). Performance of the GeneXpert Ebola assay for diagnosis of Ebola virus disease in Sierra Leone: A field evaluation study. PLoS Med..

[26] Clark D.V., Kibuuka H., Millard M., Wakabi S., Lukwago L., Taylor A., Eller M.A., Eller L.A., Michael N.L., Honko A.N. (2015). Long-term sequelae after Ebola virus disease in Bundibugyo, Uganda: A retrospective cohort study. Lancet Infect. Dis..

[27] Kaweesa R.E., Katende J.S., Wayesu R.R., Ntabadde A.D., Opio S., Kato L., Oluka G.K., Nambi R., Tumusiime R.A., FiloStudy Team (2025). Resilience and residuals beyond containment—The hidden burden of Bundibugyo Ebola virus survivorship sixteen years on: A cross-sectional observational study. New Microbes New Infect..

[28] Lewis C.E., Pinette M.M., Lakin S.M., Smith G., Fisher M., Moffat E., Embury-Hyatt C., Pickering B.S. (2024). Experimental infection of Bundibugyo virus in domestic swine leads to viral shedding with evidence of intraspecies transmission. Transbound. Emerg. Dis..

[29] Gupta M., MacNeil A., Reed Z.D., Rollin P.E., Spiropoulou C.F. (2012). Serology and cytokine profiles in patients infected with the newly discovered Bundibugyo ebolavirus. Virology.

[30] Woolsey C., Borisevich V., Agans K.N., Fenton K.A., Cross R.W., Geisbert T.W. (2021). Bundibugyo ebolavirus survival is associated with early activation of adaptive immunity and reduced myeloid-derived suppressor cell signaling. mBio.

[31] Kozak R., He S., Kroeker A., de La Vega M.A., Audet J., Wong G., Urfano C., Antonation K., Embury-Hyatt C., Kobinger G.P. (2016). Ferrets infected with Bundibugyo virus or Ebola virus recapitulate important aspects of human filovirus disease. J. Virol..

[32] Marzi A. (2025). One-for-one or one-for-all? Considerations for filovirus vaccine development. PLoS Biol..

[33] Clark D.J., Tyson J., Sails A.D., Krishna S., Staines H.M. (2018). The current landscape of nucleic acid tests for filovirus detection. J. Clin. Virol..

[34] World Health Organization (2024). Diagnostic Testing for Ebola and Marburg Virus Diseases: Interim Guidance, 20 December 2024.

[35] Kopp K., Smith I., Klein R., Todd S., Marsh G.A., Ward A.C. (2019). Pan-filovirus one-step reverse transcription-polymerase chain reaction screening assay. bioRxiv.

[36] Panning M., Laue T., Olschläger S., Eickmann M., Becker S., Raith S., Courbot M.C., Nilsson M., Gopal R., Lundkvist A. (2007). Diagnostic reverse-transcription polymerase chain reaction kit for filoviruses based on the strain collections of all European biosafety level 4 laboratories. J. Infect. Dis..

[37] Ogawa H., Miyamoto H., Ebihara H., Ito K., Morikawa S., Feldmann H., Takada A. (2011). Detection of all known filovirus species by reverse transcription-polymerase chain reaction using a primer set specific for the viral nucleoprotein gene. J. Virol. Methods.

[38] Rieger T., Kerber R., El Halas H., Pallasch E., Duraffour S., Günther S., Ölschläger S. (2016). Evaluation of RealStar reverse transcription-polymerase chain reaction kits for filovirus detection in the laboratory and field. J. Infect. Dis..

[39] Coertse J., Mortlock M., Grobbelaar A., Moolla N., Markotter W., Weyer J. (2023). Development of a Pan-Filoviridae SYBR Green qPCR Assay for Biosurveillance Studies in Bats. Viruses.

[40] Cui N., Perez Y.L., Hume A.J., Nunley B.E., Kong K., Mills M.G., Xie H., Greninger A.L. (2024). A high-throughput, polymerase-targeted RT-PCR for broad detection of mammalian filoviruses. Microbiol. Spectr..

[41] altona Diagnostics GmbH (2021). RealStar® Filovirus Screen RT-PCR Kit 1.0 Instructions for Use (Version 08/2021 EN).

[42] World Health Organization WHO EUAL Public Report: EVD IVD Product RealStar® Filovirus Screen RT-PCR Kit 1.0, EAE 0425-153-00. Amended 31 January 2019. https://extranet.who.int/prequal/key-resources/documents/who-eual-public-report-evd-ivd-product-realstarr-filovirus-screen-rt-pcr.

[43] World Health Organization (2026). Bundibugyo Virus (BDBV) Nucleic Acid Detection Tests: Ongoing EUL Applications.

[44] Amuri-Aziza A., Adroba Tandele P., Luakanda-Ndelemo G., Kinganda-Lusamaki E., Lola-Loway M., Djemba-Fundji B., Ola-Mpumbe R., Cikaya-Kankolongo F., Paku-Tshambu P., Muswamba-Kayembe P.-C. Initial Genomes from May 2026 Bundibugyo Virus Disease Outbreak in the Democratic Republic of the Congo and Uganda Reveal a New Spillover Event. Virological.org 2026. https://virological.org/t/initial-genomes-from-may-2026-bundibugyo-virus-disease-outbreak-in-the-democratic-republic-of-the-congo-and-uganda/1032.

[45] altona Diagnostics GmbH (2020). RealStar® Ebolavirus RT-PCR Kit 1.0 Instructions for Use (Version 05/2020).

[46] U.S. Food and Drug Administration RealStar Ebolavirus RT-PCR Kit 1.0 (altona Diagnostics GmbH), Emergency Use Authorization; Initial Issuance 10 November 2014, reissued 26 November 2014. https://www.fda.gov/media/123410/download?attachment.

[47] Africa Centres for Disease Control and Prevention (Africa CDC) (2026). Molecular Diagnostic Tests (RT-PCR) for Ebola Virus Bundibugyo Strain.

[48] Trombley A.R., Wachter L., Garrison J., Buckley-Beason V.A., Jahrling J., Hensley L.E., Schoepp R.J., Norwood D.A., Goba A., Fair J.N. (2010). Comprehensive panel of real-time TaqMan polymerase chain reaction assays for detection and absolute quantification of filoviruses, arenaviruses, and New World hantaviruses. Am. J. Trop. Med. Hyg..

[49] Lin X., Jin X., Xu B., Wang R., Fu R., Su Y., Jiang K., Yang H., Lu Y., Guo Y. (2019). Fast and parallel detection of four Ebola virus species on a microfluidic-chip-based portable reverse transcription loop-mediated isothermal amplification system. Micromachines.

[50] altona Diagnostics GmbH (2021). RealStar® Filovirus Type RT-PCR Kit 2.0 Instructions for Use (Version 03/2021 EN).

[51] altona Diagnostics GmbH altona Diagnostics Filovirus PCR Kits Detect Current Bundibugyo Ebolavirus Variant. https://altona-diagnostics.com/altona-diagnostics-filovirus-pcr-kits-detect-current-bundibugyo-ebolavirus-variant/.

[52] International Pandemic Preparedness Secretariat (2026). Ebola (Bundibugyo) Day 15: The Status of Diagnostics, Investigational Therapeutics and Vaccines. https://ippsecretariat.org/news/day-15-ebola-bdbv-2026/.

[53] U.S. Food and Drug Administration (2023). BioFire Global Fever Special Pathogens Panel 510(k) Summary (K213362).

[54] Collins B., Rabiger D.S., Gurling M., Helm J.R., Smith W., Belgique P., Jackson O., Johnson M., Kelley A.J., VanHollebeke H. (2023). Clinical Evaluation of the BioFire® Global Fever Special Pathogens Panel. Open Forum Infect. Dis..

[55] U.S. Food and Drug Administration (FDA) (2026). Xpert® Hemorrhagic Fever Test 510(k) Premarket Notification (Decision K253653).

[56] Cepheid Editorial Team Ebola Outbreak 2026: Closing Critical Diagnostic Gaps. https://www.cepheid.com/en-US/insights/insight-hub/community-and-global-health/2026/06/ebola-outbreak-2026-closing-critical-diagnostic-gaps.html.

[57] KH Medical Co., Ltd. (2024). RADIONE Ebola Detection Kit (Cat No. RP017) Product Manual.

[58] Wawina-Bokalanga T., Amuri-Aziza A., Vercauteren K., Mukadi-Bamuleka D., Ngandu C., Kindrachuk J., Akilimali P., Muyembe-Tamfum J.J., Mbala-Kingebeni P., Ebola Research and Response Team (2026). Diagnostic performance of RADIONE for diagnosis of BDBV. Lancet Infect. Dis..

[59] KH Medical Co., Ltd. (2024). RADIONE Pan-Ebola Genotyping & Marburg Multiplex Kit (Cat No. RP038) Product Manual.

[60] Shanghai ZJ Bio-Tech Co., Ltd. (2025). Ebola Virus (EBOV) Real Time RT-PCR Kit Instructions for Use (Product Code: QR-0220-02), Rev. ZJ0003.

[61] World Health Organization WHO Lists the First BDBV Test Under Its EUL Procedure. https://extranet.who.int/prequal/news/who-lists-first-bdbv-test-under-its-eul-procedure.

[62] World Health Organization (2026). WHO Emergency Use List: Bundibugyo Virus (BDBV) Nucleic Acid Detection Tests.

[63] World Health Organization (2020). Amended Public Report for Liferiver™—Ebola Virus (EBOV) Real Time RT-PCR Kit (EAE 0432-139-00).

[64] Bustin S.A., Benes V., Garson J.A., Hellemans J., Huggett J., Kubista M., Mueller R., Nolan T., Pfaffl M.W., Shipley G.L. (2009). The MIQE guidelines: Minimum information for publication of quantitative real-time PCR experiments. Clin. Chem..

[65] Quick J., Loman N.J., Duraffour S., Simpson J.T., Severi E., Cowley L., Bore J.A., Koundouno R., Dudas G., Mikhail A. (2016). Real-time, portable genome sequencing for Ebola surveillance. Nature.

[66] Kopp K. (2026). Whole-Genome Sequencing for High-Consequence Emerging RNA Viruses: Strategy Selection for Bundibugyo Virus Disease Under 2026 Outbreak Constraints. Viruses.

[67] International Pandemic Preparedness Secretariat (2026). Ebola (Bundibugyo) Day 45: Tracking Progress on Medical Countermeasures. https://ippsecretariat.org/news/ebola-bundibugyo-day-45-tracking-progress-on-medical-countermeasures/.

[68] World Health Organization Bundibugyo Virus Disease PHEIC: Emergency Use Listing Procedure (EUL) for IVDs. https://extranet.who.int/prequal/vitro-diagnostics/bundibugyo-virus-disease-pheic-emergency-use-listing-procedure-eul-ivds.

[69] U.S. Food and Drug Administration (2019). Evaluation of Automatic Class III Designation for OraQuick Ebola Rapid Antigen Test: Decision Summary DEN190025. https://www.accessdata.fda.gov/cdrh_docs/reviews/DEN190025.pdf.

[70] Wang Z., Bennett R.S., Roehler M., Guillon G., Fischl M.J., Donadi M.C., Makovetz J., Holmes N., Zaveri T., Toolan E. (2023). Development and clinical evaluation of a rapid point-of-care test for Ebola virus infection in humans. Viruses.

[71] Mukadi-Bamuleka D., Bulabula-Penge J., Jacobs B.K.M., De Weggheleire A., Edidi-Atani F., Mambu-Mbika F., Legand A., Klena J.D., Fonjungo P.N., Mbala-Kingebeni P. (2023). Head-to-head comparison of diagnostic accuracy of four Ebola virus disease rapid diagnostic tests versus GeneXpert® in eastern Democratic Republic of the Congo outbreaks: A prospective observational study. eBioMedicine.

[72] World Health Organization CORC Filovirus Research & Development Roadmap. https://cdn.who.int/media/docs/default-source/consultation-rdb/corc-filovirus-roadmap.pdf?download=true&sfvrsn=7dd4fa_1.

[73] Levine C.B., Mire C.E., Geisbert T.W. (2021). Comparison of Zaire and Bundibugyo ebolavirus polymerase complexes and susceptibility to antivirals through a newly developed Bundibugyo minigenome system. J. Virol..

[74] Warren T.K., Jordan R., Lo M.K., Ray A.S., Mackman R.L., Soloveva V., Siegel D., Perron M., Bannister R., Hui H.C. (2016). Therapeutic efficacy of the small molecule GS-5734 against Ebola virus in rhesus monkeys. Nature.

[75] Lo M.K., Albariño C.G., Perry J.K., Chang S., Tchesnokov E.P., Guerrero L., Chakrabarti A., Shrivastava-Ranjan P., Chatterjee P., McMullan L.K. (2020). Remdesivir targets a structurally analogous region of the Ebola virus and SARS-CoV-2 polymerases. Proc. Natl. Acad. Sci. USA.

[76] Cross R.W., Woolsey C., Chu V.C., Babusis D., Bannister R., Vermillion M.S., Geleziunas R., Barrett K.T., Bunyan E., Nguyen A.Q. (2024). Oral administration of obeldesivir protects nonhuman primates against Sudan ebolavirus. Science.

[77] Cross R.W., Woolsey C., Prasad A.N., Borisevich V., Agans K.N., Deer D.J., Harrison M.B., Dobias N.S., Fenton K.A., Cihlar T. (2025). Oral obeldesivir provides postexposure protection against Marburg virus in nonhuman primates. Nat. Med..

[78] Woolsey C., Cross R.W., Chu V.C., Prasad A.N., Agans K.N., Borisevich V., Deer D.J., Harrison M.B., Martinez J.K., Dobias N.S. (2025). The oral drug obeldesivir protects nonhuman primates against lethal Ebola virus infection. Sci. Adv..

[79] Island Pharmaceuticals Ltd Government Approvals Secured for the Use of Galidesivir as a Treatment for Bundibugyo Ebola Epidemic in Africa. ASX Announcement, 7 July 2026. https://announcements.asx.com.au/asxpdf/20260707/pdf/071g0w0swy8rq6.pdf.

[80] BiotechDispatch (2026). Uganda Approves Galidesivir for Compassionate Use Amid Bundibugyo Ebola Outbreak. https://biotechdispatch.com.au/news/uganda-approves-galidesivir-for-compassionate-use-amid-bundibugyo-ebola-outbreak.

[81] Taylor R., Kotian P., Warren T., Panchal R., Bavari S., Julander J., Dobo S., Rose A., El-Kattan Y., Taubenheim B. (2016). BCX4430: A broad-spectrum antiviral adenosine nucleoside analog under development for the treatment of Ebola virus disease. J. Infect. Public Health.

[82] Warren T., MacLennan S., Mathis A., Giuliano E., Taylor R., Sheridan W. (2017). Efficacy of galidesivir against Ebola virus disease in rhesus monkeys. Open Forum Infect. Dis..

[83] Comer J.E., Escaffre O., Neef N., Brasel T., Juelich T.L., Smith J.K., Smith J., Kalveram B., Perez D.D., Massey S. (2019). Filovirus virulence in interferon α/β and γ double knockout mice, and treatment with favipiravir. Viruses.

[84] U.S. Food and Drug Administration VEKLURY (Remdesivir) for Injection, for Intravenous Use: Prescribing Information. https://www.accessdata.fda.gov/drugsatfda_docs/label/2025/214787s030lbl.pdf.

[85] European Medicines Agency Veklury. https://www.ema.europa.eu/en/medicines/human/EPAR/veklury.

[86] Pharmaceuticals and Medical Devices Agency Avigan Tablet 200 mg: Report on the Deliberation Results. https://www.pmda.go.jp/files/000210319.pdf.

[87] Central Drugs Standard Control Organisation List of New Drugs Approved in the Year 2020 till Date. https://cdsco.gov.in/opencms/resources/UploadCDSCOWeb/2018/UploadApprovalNewDrugs/newdrugs%20approval%20july2020.pdf.

[88] Gilead Sciences Neglected and Emerging Viruses and Pandemic Preparedness. https://www.gilead.com/science/therapeutic-areas/virology/neglected-and-emerging-viruses-and-pandemic-preparedness.

[89] Prasad A.N., Woolsey C., Borisevich V., Agans K.N., Deer D.J., Geisbert J.B., Harrison M.B., Dobias N.S., Fenton K.A., Cross R.W. (2025). Remdesivir, mAb114, REGN-EB3, and ZMapp partially rescue nonhuman primates infected with a low-passage Kikwit variant of Ebola virus. Nat. Commun..

[90] Flyak A.I., Shen X., Murin C.D., Turner H.L., David J.A., Fusco M.L., Lampley R., Kose N., Ilinykh P.A., Kuzmina N. (2016). Cross-reactive and potent neutralizing antibody responses in human survivors of natural ebolavirus infection. Cell.

[91] King L.B., West B.R., Moyer C.L., Gilchuk P., Flyak A., Ilinykh P.A., Bombardi R., Hui S., Huang K., Bukreyev A. (2019). Cross-reactive neutralizing human survivor monoclonal antibody BDBV223 targets the ebolavirus stalk. Nat. Commun..

[92] Santos R.I., Ilinykh P.A., Pietzsch C.A., Ronk A.J., Huang K., Kuzmina N.A., Zhou F., Crowe J.E., Bukreyev A. (2023). Blocking of ebolavirus spread through intercellular connections by an MPER-specific antibody depends on BST2/tetherin. Cell Rep..

[93] Gilchuk P., Mire C.E., Geisbert J.B., Agans K.N., Deer D.J., Cross R.W., Slaughter J.C., Flyak A.I., Mani J., Pauly M.H. (2018). Efficacy of human monoclonal antibody monotherapy against Bundibugyo virus infection in nonhuman primates. J. Infect. Dis..

[94] Ilinykh P.A., Graber J., Kuzmina N.A., Huang K., Ksiazek T.G., Crowe J.E., Bukreyev A. (2018). Ebolavirus chimerization for the development of a mouse model for screening of Bundibugyo-specific antibodies. J. Infect. Dis..

[95] Zhang B.Y., Li F.X., Wang S., Zhou J.H., Zhou J.G., Wu Y., Xie Y., Li W.J., Yan F.H., Zhang X.H. (2026). Lethal VSV-based surrogate mouse models for Sudan and Bundibugyo ebolaviruses enable antibody evaluation under BSL-2 conditions. Zool. Res..

[96] Bornholdt Z.A., Herbert A.S., Mire C.E., He S., Cross R.W., Wec A.Z., Abelson D.M., Geisbert J.B., James R.M., Rahim M.N. (2019). A two-antibody pan-ebolavirus cocktail confers broad therapeutic protection in ferrets and nonhuman primates. Cell Host Microbe.

[97] Mulangu S., Dodd L.E., Davey R.T., Tshiani Mbaya O., Proschan M., Mukadi D., Lusakibanza Manzo M., Nzolo D., Tshomba Oloma A., Ibanda A. (2019). A randomized, controlled trial of Ebola virus disease therapeutics. N. Engl. J. Med..

[98] U.S. Food and Drug Administration INMAZEB (Atoltivimab, Maftivimab, and Odesivimab-Ebgn) Injection, for Intravenous Use: Prescribing Information. https://www.accessdata.fda.gov/drugsatfda_docs/label/2024/761169s011lbl.pdf.

[99] Rayaprolu V., Fulton B.O., Rafique A., Arturo E., Williams D., Hariharan C., Callaway H., Parvate A., Schendel S.L., Parekh D. (2023). Structure of the Inmazeb cocktail and resistance to Ebola virus escape. Cell Host Microbe.

[100] World Health Organization Sudan Ebolavirus—Experts Deliberations: Candidate Treatments Prioritization and Trial Design Discussions, October & November 2022. https://cdn.who.int/media/docs/default-source/blue-print/sudan-therapeutics-prioritization-and-trial-design-committee-summary-nov-15-2022_final-web.pdf?download=true&sfvrsn=3d04f6b6_4.

[101] Gilchuk P., Kuzmina N., Ilinykh P.A., Huang K., Gunn B.M., Bryan A., Davidson E., Doranz B.J., Turner H.L., Fusco M.L. (2018). Multifunctional pan-ebolavirus antibody recognizes a site of broad vulnerability on the ebolavirus glycoprotein. Immunity.

[102] Gilchuk P., Murin C.D., Cross R.W., Ilinykh P.A., Huang K., Kuzmina N., Borisevich V., Agans K.N., Geisbert J.B., Zost S.J. (2021). Pan-ebolavirus protective therapy by two multifunctional human antibodies. Cell.

[103] Milligan J.C., Davis C.W., Yu X., Ilinykh P.A., Huang K., Halfmann P.J., Cross R.W., Borisevich V., Agans K.N., Geisbert J.B. (2022). Asymmetric and non-stoichiometric glycoprotein recognition by two distinct antibodies results in broad protection against ebolaviruses. Cell.

[104] Keck Z.Y., Enterlein S.G., Howell K.A., Vu H., Shulenin S., Warfield K.L., Froude J.W., Araghi N., Douglas R., Biggins J. (2016). Macaque monoclonal antibodies targeting novel conserved epitopes within filovirus glycoprotein. J. Virol..

[105] Brannan J.M., He S., Howell K.A., Prugar L.I., Zhu W., Vu H., Shulenin S., Kailasan S., Raina H., Wong G. (2019). Post-exposure immunotherapy for two ebolaviruses and Marburg virus in nonhuman primates. Nat. Commun..

[106] Frei J.C., Nyakatura E.K., Zak S.E., Bakken R.R., Chandran K., Dye J.M., Lai J.R. (2016). Bispecific antibody affords complete post-exposure protection of mice from both Ebola (Zaire) and Sudan viruses. Sci. Rep..

[107] Liu G., He S., Chan M., Zhang Z., Schulz H., Cao W., Rahim M.N., Audet J., Garnett L., Wec A. (2023). A pan-ebolavirus monoclonal antibody cocktail provides protection against Ebola and Sudan viruses. J. Infect. Dis..

[108] Wirchnianski A.S., Nyakatura E.K., Herbert A.S., Kuehne A.I., Abbasi S.A., Florez C., Storm N., McKay L.G.A., Dailey L., Kuang E. (2024). Design and characterization of protective pan-ebolavirus and pan-filovirus bispecific antibodies. PLoS Pathog..

[109] Zhou J., Zhang B., Yao Y., Li F., Chen S., Wu Y., Xie Y., Guo X., Li W., Peng C. (2026). Engineered bispecific antibodies achieve broad and potent protection against multiple ebolavirus species. Emerg. Microbes Infect..

[110] Fan P., Sun B., Liu Z., Fang T., Ren Y., Zhao X., Song Z., Yang Y., Li J., Yu C. (2024). A pan-orthoebolavirus neutralizing antibody encoded by mRNA effectively prevents virus infection. Emerg. Microbes Infect..

[111] Zhang X., Liu X., Zhou J., Gao P., Pan S., Yang X., Duan H., Liao Y., Zhang F., Dong X. (2026). Computational design of broad-spectrum Ebola antibodies through framework and complementarity-determining region synergistic optimization. Research.

[112] Zhang X., Liao Y., Liu X., Zhou J., Gao P., Dong X., Pan S., Duan H., Liu J., Chi X. (2026). Nanobody targeting glycan cap confers broad orthoebolavirus neutralization. Virol. Sin..

[113] Wang M., Zhang X., Li W., Yao Y., Li E., Zhang B., Zhou J., Liu S., Gao Y., Zhu Z. (2026). A highly potent nanobody-based bispecific therapeutic provides broad-spectrum protection against ebolavirus. Nat. Commun..

[114] Flyak A.I., Kuzmina N., Murin C.D., Bryan C., Davidson E., Gilchuk P., Gulka C.P., Ilinykh P.A., Shen X., Huang K. (2018). Broadly neutralizing antibodies from human survivors target a conserved site in the Ebola virus glycoprotein HR2-MPER region. Nat. Microbiol..

[115] Murin C.D., Gilchuk P., Crowe J.E., Ward A.B. (2022). Structural biology illuminates molecular determinants of broad ebolavirus neutralization by human antibodies for pan-ebolavirus therapeutic development. Front. Immunol..

[116] West B.R., Moyer C.L., King L.B., Fusco M.L., Milligan J.C., Hui S., Saphire E.O. (2018). Structural basis of pan-ebolavirus neutralization by a human antibody against a conserved, yet cryptic epitope. mBio.

[117] Schoeder C.T., Gilchuk P., Sangha A.K., Ledwitch K.V., Malherbe D.C., Zhang X., Binshtein E., Williamson L.E., Martina C.E., Dong J. (2022). Epitope-focused immunogen design based on the ebolavirus glycoprotein HR2-MPER region. PLoS Pathog..

[118] Mire C.E., Geisbert J.B., Marzi A., Agans K.N., Feldmann H., Geisbert T.W. (2013). Vesicular stomatitis virus-based vaccines protect nonhuman primates against Bundibugyo ebolavirus. PLoS Negl. Trop. Dis..

[119] Falzarano D., Feldmann F., Grolla A., Leung A., Ebihara H., Strong J.E., Marzi A., Takada A., Jones S., Gren J. (2011). Single immunization with a monovalent vesicular stomatitis virus-based vaccine protects nonhuman primates against heterologous challenge with Bundibugyo ebolavirus. J. Infect. Dis..

[120] Woolsey C., Borisevich V., Agans K.N., O’Toole R., Fenton K.A., Harrison M.B., Prasad A.N., Deer D.J., Gerardi C., Morrison N. (2023). A highly attenuated panfilovirus VesiculoVax vaccine rapidly protects nonhuman primates against Marburg virus and 3 species of Ebola virus. J. Infect. Dis..

[121] Kimble J.B., Malherbe D.C., Meyer M., Gunn B.M., Karim M.M., Ilinykh P.A., Iampietro M., Mohamed K.S., Negi S., Gilchuk P. (2019). Antibody-mediated protective mechanisms induced by a trivalent parainfluenza virus-vectored ebolavirus vaccine. J. Virol..

[122] Malherbe D.C., Kimble J.B., Atyeo C., Fischinger S., Meyer M., Cody S.G., Hyde M., Alter G., Bukreyev A. (2023). A single-dose intranasal combination panebolavirus vaccine. J. Infect. Dis..

[123] Felgner J., Clarke E., Hernandez-Davies J.E., Jan S., Wirchnianski A.S., Jain A., Nakajima R., Jasinskas A., Strahsburger E., Chandran K. (2024). Broad antibody and T cell responses to Ebola, Sudan, and Bundibugyo ebolaviruses using mono- and multi-valent adjuvanted glycoprotein vaccines. Antivir. Res..

[124] Lee Y.Z., Zhang Y.N., Newby M.L., Ward G., Braz Gomes K., Auclair S., DesRoberts C., Allen J.D., Ward A.B., Stanfield R.L. (2025). Rational design of next-generation filovirus vaccines combining glycoprotein stabilization and nanoparticle display with glycan modification. Nat. Commun..

[125] Singh K., Marasini B., Chen X., Ding L., Wang J.J., Xiao P., Villinger F., Spearman P. (2020). A bivalent, spherical virus-like particle vaccine enhances breadth of immune responses against pathogenic Ebola viruses in rhesus macaques. J. Virol..

[126] Xu D., Powell A.E., Utz A., Sanyal M., Do J., Patten J.J., Moliva J.I., Sullivan N.J., Davey R.A., Kim P.S. (2024). Design of universal Ebola virus vaccine candidates via immunofocusing. Proc. Natl. Acad. Sci. USA.

[127] Zhang J., Zhang X., Yao Y., Zhou J., Chen D., Shen Y., Gong Q., Li Z., Zhou Z., Li E. (2026). Multivalent mRNA vaccine platform with compatible antigens conferred broad-spectrum protection against orthoebolaviruses’ exposure. Proc. Natl. Acad. Sci. USA.

[128] CEPI CEPI Fast-Tracks Three Bundibugyo Ebolavirus Vaccine Candidates. https://cepi.net/cepi-fast-tracks-three-bundibugyo-ebolavirus-vaccine-candidates.

[129] University of Oxford Oxford Bundibugyo Ebolavirus Vaccine Candidate Receives CEPI Backing. https://www.ox.ac.uk/news/2026-06-01-oxford-bundibugyo-ebolavirus-vaccine-candidate-receives-cepi-backing.

[130] CEPI Ambitious Research to Develop Multivalent Vaccines Against Multiple Deadly Filoviruses. https://cepi.net/ambitious-research-develop-multivalent-vaccines-against-deadly-filoviruses.

[131] Woolsey C., Strampe J., Fenton K.A., Agans K.N., Martinez J., Borisevich V., Dobias N.S., Deer D.J., Geisbert J.B., Cross R.W. (2023). A recombinant vesicular stomatitis virus-based vaccine provides postexposure protection against Bundibugyo ebolavirus infection. J. Infect. Dis..

[132] Regeneron Pharmaceuticals Regeneron’s Ebola Antibody Recommended by World Health Organization for Investigational Use in Response to Current Bundibugyo Ebolavirus Outbreak. https://investor.regeneron.com/news-releases/news-release-details/regenerons-ebola-antibody-recommended-world-health-organization.

[133] Charité—Universitätsmedizin Berlin Patient Discharged from Charité’s Specialized Isolation Unit. https://www.charite.de/en/service/press_reports/artikel/detail/patient_discharged_from_charites_special_isolation_unit.

[134] European Medicines Agency EMA, AMA and African Regulatory Authorities Join Forces on Ebola Outbreak Response. https://www.ema.europa.eu/en/news/ema-ama-african-regulatory-authorities-join-forces-ebola-outbreak-response.

[135] WHO Regional Office for Africa Democratic Republic of the Congo Confirms New Ebola Outbreak, WHO Scales up Support. https://www.afro.who.int/countries/democratic-republic-of-congo/news/democratic-republic-congo-confirms-new-ebola-outbreak-who-scales-upsupport.

[136] U.S. Food and Drug Administration ERVEBO (Ebola Zaire Vaccine, Live): Package Insert. https://www.fda.gov/media/133748/download?attachment=.

